# Inflorescence architecture and development in the megadiverse *Eugenia* (Myrteae, Myrtaceae): an evolutionary perspective

**DOI:** 10.1093/aobpla/plag037

**Published:** 2026-08-20

**Authors:** Augusto Giaretta, Thais N C Vasconcelos, Marcelo T Kubo, Gerhard Prenner, Fiorella F Mazine, Jair E Q Faria, Jefferson N Fregonezi, Paulo T Sano, Eve J Lucas

**Affiliations:** Herbário DDMS, Faculdade de Ciências Biológicas e Ambientais, Universidade Federal da Grande Dourados, Unidade II, Rodovia Dourados-Itahum, Km 12, 79804-970, Caixa Postal 364, Dourados, MS, Brazil; Herbário VIC, Departamento de Biologia Vegetal, Universidade Federal de Viçosa, Av. Purdue s/n, 36570-900, Viçosa, MG, Brazil; Department of Ecology and Evolutionary Biology, University of Michigan, 1105 N University Ave, Ann Arbor, MI 48109, United States; Laboratório de Sistemática Vegetal, Instituto de Biociências, Universidade de São Paulo, Rua do Matão, 277, 05508-090, São Paulo, SP, Brazil; Sportmittelschule St. Pölten, Dr. Theodor Körner 1, Johann Gasser Strasse 7, St. Pölten 3100, Austria; Universidade Federal de São Carlos, Campus Sorocaba, Rodovia João Leme dos Santos, km 110 (SP-264), 18052-780, Sorocaba, SP, Brazil; Instituto Interamericano de Cooperação para a Agricultura – IICA, SHIS QI 5, Chácara 16, Lago Sul, 71600-530, Brasília, DF, Brazil; Herbário VIC, Departamento de Biologia Vegetal, Universidade Federal de Viçosa, Av. Purdue s/n, 36570-900, Viçosa, MG, Brazil; Laboratório de Sistemática Vegetal, Instituto de Biociências, Universidade de São Paulo, Rua do Matão, 277, 05508-090, São Paulo, SP, Brazil; Royal Botanic Gardens, Kew, Herbarium K, Richmond, Surrey TW9 3AB, United Kingdom

**Keywords:** Eugeniinae, *Eugenia*, Myrteae, evolution, ontogeny, homoplasy, inflorescence scaffolding

## Abstract

This work explores inflorescence development patterns in *Eugenia* (Myrtaceae), one of the largest genera of woody plants with over 1200 species. The central hypothesis is that all variation in inflorescence arrangements in *Eugenia* is homologous. This is addressed as specific aims: (i) to identify the main inflorescence developmental patterns, (ii) to test the homology of inflorescence architectures using a time-calibrated phylogeny, and (iii) to assess and update traditional terminology. Taxonomically representative samples of *Eugenia* inflorescences were analysed to provide an evolutionary and comparative framework. Comparative ontogenetic analysis incorporated 24 taxa, while mature material analysis encompassed 66 species included in a dated phylogeny. Racemose branching is the ancestral condition of *Eugenia*. Seven development inflorescence patterns were recognized and combined into five patterns (*auxotelic* + *auxotelic cataphylls*, *fasciculiform* + *glomeruliform*, *umbelliform*, *racemiform*, and *thyrsoid*) to focus on evolutionary traits. The *auxotelic* pattern is the ancestral condition of *Eugenia* recovered in the early nodes and in the backbone with the highest probabilities of the reconstructed phylogeny, whereas *auxotelic* and *fasciculiform* were the most recurrent patterns in *Eugenia* sect. *Umbellatae*. The relative length and number of main axes that arise from an axil are shown to be systematically relevant, while *racemiform* pattern is exclusive to *Eugenia* sect. *Racemosae*. Flexible inflorescence arrangements found in *Eugenia* reflect a continuous spectrum of morphologies derived from a common ancestral scaffold. The combination of intense floral display in the *fasciculiform* pattern and flexibility may represent a key innovation in *E*. sect. *Umbellatae*. This work highlights the evolutionary significance of inflorescences and improves existing terminology.

## Teaser

This study shows that the rich diversity of inflorescences in *Eugenia* (myrtle family) is built from a small set of shared developmental patterns derived from an ancestrally racemose scaffold. By combining ontogeny, mature morphology, and time-calibrated phylogenies, we demonstrate that these architectures form a continuous spectrum rather than independent types, and we propose an updated, evolutionarily informed terminology that improves taxonomy and systematics in Myrtaceae.

## Introduction

Improved understanding of phylogenetic relationships among flowering plants has stimulated interest in comparative morphological and developmental analyses of inflorescences, defined as the arrangement of flowers along the reproductive axis of angiosperms (Stebbins 1973, Bull-Hereñu and Claßen-Bockhoff 2011, Kirchoff and Claßen-Bockhoff 2013). This has led to renewed interpretations of the functional and evolutionary significance of inflorescence architecture (Benlloch *et al*. 2007, Prenner *et al*. 2009, Castel *et al*. 2010, Claßen-Bockhoff and Bull-Hereñu 2013). Inflorescence architecture is highly flexible in many angiosperm groups (Tooke *et al*. 2005, Harder and Prusinkiewicz 2013), generating numerous forms that have traditionally been classified based on mature structures (e.g. Rickett 1944, Weberling 1988, Troll and Weberling 1989). However, environmental and genetic constraints shaping these architectures remain poorly explored (Prusinkiewicz et al. 2007, Kirchoff and Claßen-Bockhoff 2013) or how widely variable and flexible mature arrangements may reflect external stimuli rather than evolutionary relationship, contributing to terminological inconsistencies and ongoing confusion and constant need for definitions for exceptional arrangements in inflorescence classification.

Inflorescence architecture is highly variable in Myrtaceae, a large angiosperm family of ca. 6000 species distributed in the Paleotropics and Neotropics (Berg 1857, POWO 2025). Briggs and Johnson (1979) produced a seminal study of Myrtaceae inflorescences based on mature architectures, integrating morphology and phylogeny across several genera. However, their emphasis on mature arrangements resulted in an incomplete framework and in a daunting terminology. A more comprehensive approach incorporating character reconstruction is needed to clarify evolutionary relationships and homologies, particularly in Myrteae, where parallel evolution is documented (Giaretta et al. 2019b, Vasconcelos *et al*. 2019) and the risk of misinterpretation as a consequence of homoplasy (Wake *et al*. 2011) is high. In this sense, assessment of homologies at lower ranks is assumed to be more accurate and less prone to misinterpretations.

About half of Myrtaceae diversity occurs in the monophyletic tribe Myrteae (ca. 2700 species, mostly Neotropical; Lucas *et al*. 2019), which exhibits marked flexibility in inflorescence morphology (see Weberling 1988). Nevertheless, a general model for Neotropical species has been proposed based on qualitative analyses (see McVaugh 1956, 1968). Following the terminology of Hallé (2001) and Chomicki *et al.* (2017), Neotropical Myrteae show axillary, sympodial modular growth as seasonal shoot production, in which successive growth units arise from axillary meristems as previous modules become determinate often by bearing inflorescences or ceasing extension growth. Growth is typically orthotropic, with opposite, decussate leaves, and some species develop protective cataphyll involucres associated with growth units (Landrum and Kawasaki 1997, Wilson 2011, Lucas *et al*. 2018).

Traditional classifications of Myrtaceae inflorescences rely on the detailed and evolutionary framework of Briggs and Johnson (1979), later simplified for Myrteae by Landrum and Kawasaki (1997). Subsequently, studies have applied diverse and non-standardized terminologies, often to identical structures (see Kubo et al. 2025 for a review of concepts and terminologies used for Myrteae inflorescence). To avoid redundancy and simplify terminology, this study adopts a descriptive approach rather than a single classificatory scheme, based on three common growth processes: branching, elongation, and reiteration. Branching is either racemose or cymose (Endress 2010), elongation relates to axis extension for spatial display, and reiteration involves repetition of total or partial architectural units (Barthélémy 1991). Common inflorescence patterns in Myrteae and *Eugenia* are briefly described following previous studies (e.g. McVaugh 1956, 1968, Briggs and Johnson 1979, Weberling 1988, Barroso *et al*. 1991, Landrum and Kawasaki 1997) and summarized in Supplementary Table S1.

**Figure 1:**
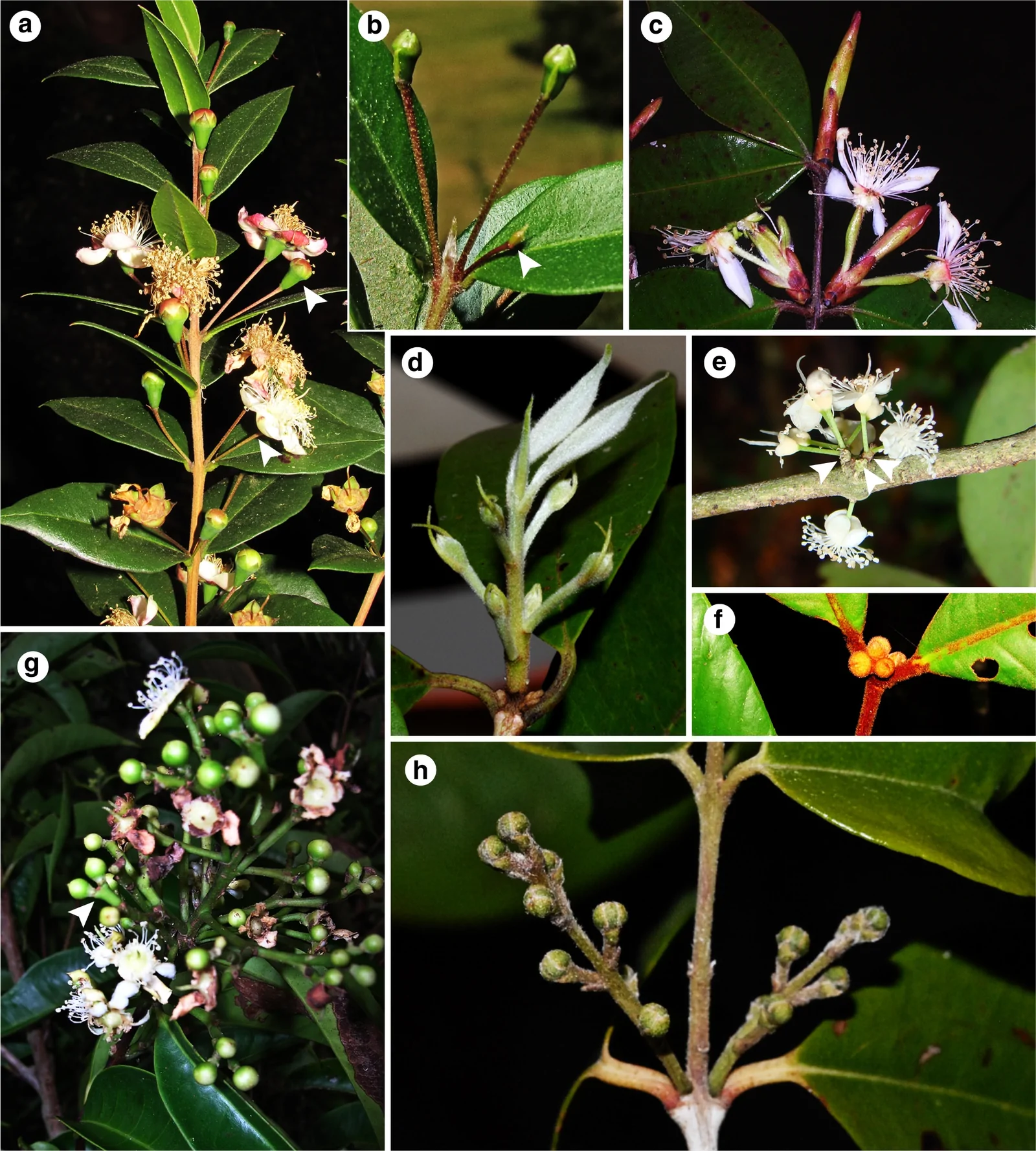
Inflorescences of *Eugenia* and *Myrtus*: (a, b) axillary flowers in *Myrtus communis* with flower reiteration shown by arrowheads; (c) auxotelic inflorescence with cataphylls in *Eugenia neosilvestris*; (d) auxotelic inflorescence in *Eugenia acutata*; (e) fasciculiform inflorescences axes shown by arrowheads in *Eugenia astringens*; (f) glomeruliform inflorescence in *Eugenia neograndifolia*; (g) thyrsoid inflorescence with a dichasial arrangement shown by an arrowhead in *Eugenia guanabarina*; (h) racemiform inflorescence in *Eugenia biflora*. Photographs taken by A.G. (a, b, c, e, f, g) and T.N.C.V. (d, h).

All these inflorescence architectures, except the panicle, can be found in *Eugenia* (Fig. 1), a genus with more than 1200 species (Mazine *et al.* 2018, POWO 2025) within subtribe Eugeniinae (Lucas *et al.* 2019). Variation in *Eugenia* inflorescence architectures is one of the highest in the family, making the genus a highly appropriate model for homology and evolutionary assessment. An obstacle to studying *Eugenia* inflorescences, however, are high levels of terminological inflation that hamper accuracy and result in the application of different terms to arrangements that repeat in different ways and at variable levels of hierarchy (Kubo *et al.* 2025, see umbelliform and racemiform in Villarroel *et al.* 2016 and Giaretta and Sano 2019, respectively; compound dichasia and flowered cyme in Faria and Fernandes 2020 and Snow *et al.* 2012, see panicle of racemes in Mazine and Souza 2010). In this context, focus on the development of reproductive structures in *Eugenia* is highly desirable to better categorize the array of inflorescence phenotypes in the genus and to contribute to a broader understanding of inflorescence diversity within tribe Myrteae. This is addressed as three specific aims: (i) to identify the main developmental patterns in *Eugenia* and allied genera, (ii) to assess seemingly homologous structures in light of current evolutionary understanding through assessment of ancestral character reconstructions, and (iii) to review traditional terminology of inflorescences in *Eugenia* and update where appropriate.

## Materials and methods

The survey included all 11 infrageneric groups of *Eugenia* (for the number of species assigned to each section of *Eugenia,* see Mazine *et al.* 2016, 2018), i.e. *Eugenia* sects. *Pseudeugenia* (3 species surveyed/14% of the total number of species in the section), *Hexachlamys* (2/20%), *Eugenia* (3/10%), *Pilothecium* (3/14%), *Jossinia* (3/1.5%), *Racemosae* (4/7%), *Phyllocalyx* (3/19%), *Excelsae* (3/60%), *Schizocalomyrtus* (6/60%), *Speciosae* (3/50%), and *Umbellatae* (28/4%). This sampling comprised a wide range of inflorescence morphological variation and provides a representative taxonomic and morphological framework for the downstream analyses.

### Survey of inflorescence development patterns

The survey encompassed two activities focused on different reproductive phases of a species. First, a general survey of inflorescence development based on material at different developmental stages, including both herbarium specimens and freshly collected samples preserved in 70% ethanol, was undertaken to recognize common patterns and those specific to the sections of *Eugenia*. Then, a broader survey of mature inflorescences was undertaken to better appreciate the flexibility of arrangements and to provide greater evolutionary and comparative perspective on the genus. Descriptions of developmental stages and mature structures run from base to apex. Additionally, panicle is considered as a comparative reference to the outgroup *Myrcia tomentosa* and is not treated as a developmental pattern within *Eugenia*. Therefore, panicle is not illustrated or analysed in detail together with the other patterns.

#### Comparative ontogenetic analysis

Inflorescence material collected in different development stages was examined from the spirit collection (70% alcohol) at the Royal Botanic Gardens, Kew. In total, 33 samples of 24 taxa were selected for comparative ontogenetic analysis using scanning electron microscopy (SEM) to assess morphological patterns observed in the mature material (Table 1). A developmental pattern was assigned to each section of *Eugenia* (*sensu* Mazine *et al.* 2018) when a given pattern was found to be fixed in two or more species (where available) in that section. For comparative purposes, the following relevant traits were recorded: meristem position (e.g. axillary or apical), developmental sequence (e.g. from main axis to lateral branching), degree of extension (e.g. relative length of successive internodes in an axis), apical growth (e.g. continued or latent) and accessory flowering axes (e.g. flowering axes sharing an axil in delayed or equivalent development stages). Accessory units are the result of more than one axillary meristem at a single leaf axil (Barthélémy and Caraglio 2007) that can produce flowers or flowering axes in different development stages (see Figs 1b and 4d).

**Table 1:** Herbarium vouchers used in the ontogenetic and molecular analyses, DNA regions, GenBank accession numbers, and country.

Species	Collector	Number	Herbarium	ITS	psbA-trnH	rpl16	rpl32-trnL	trnQ-rps16	Country
***Eugenia acutata* Miq.**	T. Vasconcelos	506	K	MF954031	MF954288	MF954331	MF954216	MF954095	Brazil
*Eugenia arvensis* Vell.	A. Brandão	159	RBR	MN296361	MN887358	MN887388	MN887418	MN629363	Brazil
***Eugenia angustissima* O.Berg**	T. Vasconcelos	278	K	–	–	–	–	–	Brazil
***Eugenia angustissima* O.Berg**	T. Vasconcelos	405	K	–	–	–	–	–	Brazil
*Eugenia astringens* Cambess.	F. Mazine	782	ESA, K	KJ187606	KJ469655	MH446113	PP135397	MH446296	Brazil
*Eugenia aurata* O.Berg	V. Staggemeier	315	K	PP002368	PP236810	PP158515	PP135327	PP131234	Brazil
*Eugenia axillaris* (Sw.) Willd.	M. Hamilton	553	K	KJ187607	KJ469656	MH446132	MH446225	MH446315	Turks & Caicos
*Eugenia azuruensis* O.Berg	J.E.Q. Faria	4186	UB	MF954033	MF954290	MF954333	MF954423	–	Brazil
***Eugenia belemitana* McVaugh**	A. Giaretta	1441	K	MF954042	MF954299	MF954342	MF954219	MF954105	Brazil
*Eugenia biflora* (L.) DC.	F. Mazine	1075	ESA	KJ187610	KJ469659	PP208972	PP135443	PP158433	Brazil
***Eugenia biflora* (L.) DC.**	T. Vasconcelos	589	K	–	–	–	–	–	Brazil
***Eugenia biflora* (L.) DC.**	A. Giaretta	1531	K	–	–	–	–	–	Brazil
*Eugenia bimarginata* DC.	F. Mazine	469	ESA, K	KJ187611	KJ469660	MH446106	MH446199	MH446289	Brazil
*Eugenia brasiliensis* Lam.	E. Lucas	126	K	KJ187613	KJ469662	MH446120	PP135445	MH446303	Brazil
*Eugenia bullata* Pancher ex Guillaumin	T. Vasconcelos	608	K	MF954034	MF954291	MF954334	MF954424	MF954097	New Caledonia
***Eugenia bunchosiifolia* Nied.**	T. Vasconcelos	466	K	MF954041	MF954298	MF954341	MF954218	MF954104	Brazil
***Eugenia candolleana* DC.**	T. Vasconcelos	s/n	K	–	–	–	–	–	Brazil
*Eugenia cerasiflora* Miq.	T. Vasconcelos	419	K, UB	MH446019	MH446077	MH446170	MH446261	MH446353	Brazil
*Eugenia coffeifolia* DC.	B. Holst	9516	SEL	MH445992	MH446049	MH446141	MH446234	MH446324	Brazil
***Eugenia coffeifolia* DC.**	A. Giaretta	1592	K	–	–	–	–	–	French Guiana
*Eugenia dodonaeifolia* Cambess.	E. Lucas	257	ESA, K	KJ187644	KJ469693	MH446123	MH446216	MH446306	Brazil
*Eugenia dysenterica* DC.	F. Mazine	466	ESA, K	KJ187620	KJ469669	KJ469669	PP135394	MH446297	Brazil
***Eugenia dysenterica* DC.**	J.E.Q. Faria	6364	K	–	–	–	–	–	Brazil
***Eugenia dysenterica* DC.**	J.E.Q. Faria	6480	K	–	–	–	–	–	Brazil
*Eugenia egensis* DC.	T. Vasconcelos	319	K	MH446026	MH446084	MH446177	MH446268	MH446360	Brazil
*Eugenia excelsa* O.Berg	E. Lucas	125	ESA, K	KJ187621	KJ469670	MH446121	PP135452	MH446304	Brazil
*Eugenia expansa* (O.Berg) Nied.	M. Bünger	634	BHCB, K	KX789279	KX789297	KX789322	KX789351	KX910672	Brazil
*Eugenia flavescens* DC.	D. Zappi	415	K	MH445982	MH446039	MH446097	MH446189	MH446279	Brazil
*Eugenia florida* DC.	F. Mazine	965	ESA, K	KJ187622	KJ469671	MH446112	MH446205	MH446295	Brazil
*Eugenia foetida* Pers.	B. Holst	8865	Cultivated SEL	MH445997	MH446054	MH446146	MH446239	MH446329	Unknown
*Eugenia goiapabana* Sobral & Mazine	M. Bünger	s/n	BHCB	KX789270	KX789300	KX789325	KX789354	KX910675	Brazil
***Eugenia guanabarina* Giaretta & M.C.Souza**	A. Giaretta	1629	K, SPF	MN296379	MN887376	MN887406	MN887436	MN629381	Brazil
***Eugenia guanabarina* Giaretta & M.C.Souza**	A. Giaretta	1630	K	–	–	–	–	–	Brazil
*Eugenia handroi* Mattos	F. Mazine	951	ESA, K	KJ187654	KJ469704	MH446104	PP135456	MH446287	Brazil
*Eugenia involucrata* DC.	M. Bünger	551	BHCB	KX789281	KX789294	KX789319	KX789348	KX910669	Brazil
***Eugenia involucrata* DC.**	J.E.Q. Faria	4047	K	–	–	–	–	–	Brazil
*Eugenia joseramosii* M.A.D. Souza & Scudeller	A. Giaretta	1655	SPF, K	MN296383	MN887380	MN887410	MN887440	MN629385	Brazil
*Eugenia lagoensis* Kiaersk.	C. Fraga	2436	K	MN296386	MN887383	MN887413	MN887443	MN629388	Brazil
***Eugenia ligustrina* (Sw.) Willd.**	T. Vasconcelos	570	K	–	–	–	–	–	Brazil
*Eugenia melanogyna* (D.Legrand) Sobral	F. Mazine	969	ESA, K	KJ187624	KJ469673	PP208984	PP135447	MH446284	Brazil
*Eugenia modesta* DC.	F. Mazine	854	ESA, K	KJ187625	–	MH446108	MH446201	MH446291	Brazil
***Eugenia modesta* DC.**	T. Vasconcelos	476	K	–	–	–	–	–	Brazil
*Eugenia monticola* (Sw.) DC.	T. Vasconcelos	566	K	MF954037	MF954294	MF954337	MF954427	MF954100	Dominican Republic
*Eugenia moschata* (Aubl.) Nied. ex T. Durand & B.D. Jacks.	M. Simon	2032	CEN	MN296385	MN887382	MN887412	MN887442	MN629387	Brazil
*Eugenia myrcianthes* Nied.	J.E.Q. Faria	2850	UB	MH446003	MH446060	MH446152	PP135458	MH446335	Brazil
***Eugenia myrcianthes* Nied.**	J.E.Q. Faria	6547	K	–	–	–	–	–	Brazil
*Eugenia neograndifolia* Mattos	A. Giaretta	1615	SPF	MN296377	MN887374	MN887404	MN887434	–	French Guiana
***Eugenia neograndifolia* Mattos**	A. Giaretta	1616	K	–	–	–	–	–	French Guiana
*Eugenia neolatifolia* Sobral & M.A.D.Souza	A. Giaretta	1587	K, SPF	MN296376	MN887373	MN887403	MN887433	MN629378	French Guiana
*Eugenia neoverrucosa* Sobral	E. Lucas	118	ESA, K	KJ187628	KJ469676	PP208985	MH446215	MH446305	Brazil
*Eugenia nutans* O.Berg	E. Lucas	281	ESA, K	KJ187629	KJ469677	MH446105	MH446198	MH446288	Brazil
***Eugenia paludosa* Pancher ex Brongn. & Gris**	T. Vasconcelos	646	K	MF954038	MF954295	MF954338	MF954428	MF954101	New Caledonia
*Eugenia patens* Poir.	E. Lucas	104	ESA, K	KJ187633	K20947	KJ469681	KX789361	KX910681	French Guiana
*Eugenia pisiformis* Cambess.	E. Lucas	232	ESA, K	KJ187634	KJ469682	MH446119	MH446212	MH446302	Brazil
*Eugenia pistaciifolia* DC.	J.E.Q. Faria	1782	UB	MH446001	MH446058	MH446150	PP135398	MH446333	Brazil
*Eugenia pitanga* (O.Berg) Nied.	F. Mazine	1044	ESA, K	KJ187635	KJ469683	–	–	–	Brazil
*Eugenia pluriflora* DC.	F. Mazine	961	ESA, K	KJ187636	KJ469684	MH446107	MH446200	MH446290	Brazil
***Eugenia pohliana* DC.**	J.E.Q. Faria	4184	K	–	–	–	–	–	Brazil
***Eugenia protenta* DC.**	T. Vasconcelos	350	K	MH446029	MH446087	MH446179	–	MH446363	Brazil
***Eugenia protenta* DC.**	A. Giaretta	1433	K	–	–	–	–	–	Brazil
*Eugenia punicifolia* (Kunth) DC.	F. Mazine	1065	ESA, K	KJ187638	KJ469686	KX789332	KX789361	KX910682	Brazil
*Eugenia pyrifera* Faria & Proença	J.E.Q. Faria	3870	UB	PP035947	PP230747	PP208980	PP135403	PP158441	Brazil
*Eugenia pyriformis* Cambess.	F. Mazine	1028	ESA, K	KJ187639	KJ469687	MH446117	PP135423	MH446300	Brazil
*Eugenia reinwardtiana* (Blume) DC.	Hyland	9245	Cultivated QRS	AY487301	–	AY463131	–	–	Australia
*Eugenia selloi* B.D.Jacks.	M. Bünger	566	BHCB, RB	KX789278	KX789308	KX789334	KX789363	KX910684	Brazil
***Eugenia sonderiana* O.Berg**	T. Vasconcelos	735	K	PZ578092	PZ564395	PZ564396	PZ564397	PZ564398	Brazil
***Eugenia sonderiana* O.Berg**	T. Vasconcelos	740	K	–	–	–	–	–	Brazil
*Eugenia speciosa* Cambess.	M. Bünger	585	BHCB	KX789274	KX789310	KX789336	KX789365	KX910686	Brazil
***Eugenia splendens* O.Berg**	J.E.Q. Faria	4196	K	–	–	–	–	–	Brazil
***Eugenia stipitata* McVaugh**	T. Vasconcelos	677	K	MF954043	MF954300	MF954343	MF954220	–	Brazil
*Eugenia subglomerata* (Kuntze) Sobral	F. Mazine	461	ESA, K	KJ187626	KJ469674	MH446116	MH446209	MH446299	Brazil
*Eugenia subterminalis* DC.	F. Mazine	974	K	PP035967	KJ469696	MH446139	PP135449	MH446322	Brazil
*Eugenia supraaxillaris* Spring ex Mart.	F. Mazine	994	ESA, K	KJ187618	KJ469667	MH446100	PP135413	MH446282	Brazil
*Eugenia tenuipedunculata* Kiaersk.	A. Giaretta	1493	K, SPF	MN296371	MN887368	MN629373	MN887428	MN887398	Brazil
*Eugenia umbrosa* O.Berg	A. Giaretta	1498	K, SPF	MN296372	MN887369	MN887399	MN887429	MN629374	Brazil
*Eugenia uniflora* L.	E. Lucas	207	Cultivated K	AM234088	AM489828	AF215627	–	KP722202	Brazil
***Eugenia uniflora* L.**	T. Vasconcelos	s/n	Cultivated K	–	–	–	–	–	Brazil
***Eugenia uniflora* L.**	J.E.Q. Faria	6294	K	–	–	–	–	–	Brazil
*Eugenia vattimoana* Mattos	A. Giaretta	1465	K, SPF	MN296368	MN887365	MN887395	MN887425	MN629370	Brazil
*Eugenia verticillata* (Vell.) Angely	Duarte	s/n (ESA85678)	ESA, K	KJ187650	KJ469700	MH446181	MH446272	MH446365	Brazil
*Eugenia wentii* Amshoff	B. Holst	9421	K	KJ187651	K35646	KJ469701	KX789368	KX910689	French Guiana
*Myrcia tomentosa* (Aubl.) DC.	Savassi	s/n (ESA85681)	K	MH445983	MH446040	MH446103	MH446196	MH446286	Brazil
*Myrcianthes fragrans* (Sw.) McVaugh	M. Hamilton	552	K	MH445989	MH446046	MH446131	PP135453	MH446314	Turks & Caicos
***Myrcianthes fragrans* (Sw.) McVaugh**	T. Vasconcelos	s/n	Cultivated K	–	–	–	–	–	Unknown
*Myrcianthes pungens* (O.Berg) D.Legrand	J.E.Q. Faria	2759	UB	MH446002	MH446059	MH446151	PP135446	MH446334	Brazil
***Myrcianthes pungens* (O.Berg) D.Legrand**	J.E.Q. Faria	4277	K						
*Myrtus communis* L.	E. Lucas	211	Cultivated K	AM234149	AM489872	PP158468	PP135392	KP722221	Unknown
***Myrtus communis* L.**	T. Vasconcelos	s/n	Cultivated K	–	-	–	–	–	Unknown
***Myrtus communis* L.**	A. Giaretta	s/n	Cultivated K	–	–	–	–	–	Unknown
*Rhodamnia rubescens* (Benth.) Miq.	S. Belsham	83	Cultivated OTA	AM234127	AM489879	–	–	–	Australia

Names in bold indicate samples used for ontogenetic analysis.

#### Comparative analysis of mature morphology

The mature inflorescence of a total of 333 samples from 66 taxa was surveyed. The 66 analysed taxa correspond to those included in the phylogenetic reconstruction. A minimum of five specimens of each species were further examined using a stereomicroscope of the mature inflorescence (Supplementary Table S2). Specimens from the Kew herbarium (K), with complementary material from MBM, RB, SPF, and CAY herbaria (acronyms according to Thiers 2025) were examined and development patterns were noted, recording the most common arrangement for each phylogenetic tip. Brief descriptions linking ultimate architectures to their respective development patterns were also made to allow recognition of patterns from mature material.

### Terminology

**Figure 2:**
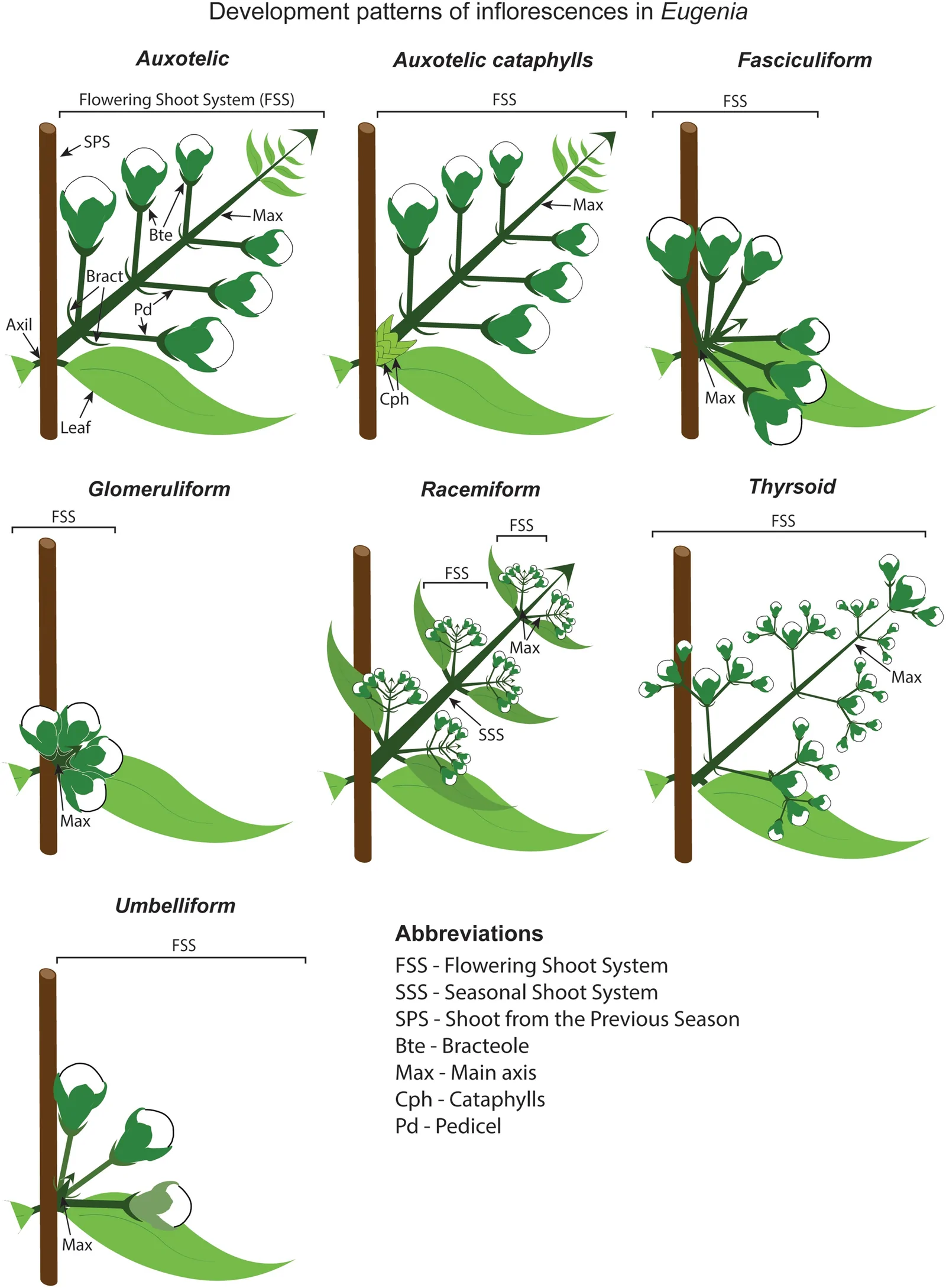
Inflorescence development pattern in *Eugenia* comparative flowering shoot systems (FSS) of described patterns at maturity.

A seasonal shoot system (henceforward SSS) is here used to refer to a shoot or a network of shoots produced in a single growing season that emerged from a shoot of a previous growing season (SPS), supporting vegetative meristems at terminal and axillary positions. These terms correspond to the SSS of Claßen-Bockhoff and Bull-Hereñu (2013) and seasonal growth unit of Briggs and Johnson (1979). Flowering shoot system (henceforward FSS) is adopted when an SSS arrives at a reproductive stage, i.e. flanked by flowers. Some vegetative meristems become reproductive, whereas others continue vegetative growth or cease. These terms (SSS, FSS and SPS) are helpful to refer to an assemblage of units in a flexible system. A brief guide to the terminology used to describe inflorescence patterns is provided (Fig. 2; also see Supplementary Table S1).

### Developmental analysis

Reproductive and vegetative material was dehydrated in alcohol series and left overnight in 100% ethanol. Samples were critically point-dried in an Autosamdri-815B critical-point dryer (Tousimis Research, Rockville, Maryland, USA). Dried material was mounted onto aluminium stubs, coated with platinum using a Quorum Q-150-T sputter coater (Quorum Technologies, East Grinstead, UK) and examined in detail using Hitachi cold field emission SEM S-4700-II (Hitachi High Technologies, Tokyo, Japan). Different stages of development of the same species available from different collections were surveyed when necessary. A total of 1092 images were generated and analysed.

### Traits reconstruction

The resulting maximum clade credibility (MCC) time-calibrated phylogeny was used to perform ancestral trait reconstruction. The function ‘make.simmap’ available in the package *phytools* v1.9-16 (Revell 2012) implemented in R (R Core Team 2025) was used to recover probabilities of ancestral character state of the nodes using a Bayesian stochastic character mapping analysis (Huelsenbeck *et al.* 2003). The inflorescences of the terminal taxa were coded first according to branching pattern (i.e. racemose, cymose, or paniculate), and then as one of seven development patterns (*auxotelic*, *auxotelic cataphylls, fasciculiform*, *glomeruliform, racemiform*, *thyrsoid*, *umbelliform*) and panicle (see Supplementary Figure S1), or a combination of five patterns (i.e. *auxotelic* + *auxotelic cataphylls*, *fasciculiform* + *glomeruliform, racemiform*, *thyrsoid*, *umbelliform*) and panicle (see Fig. 2). The combined approach with five patterns and panicle was chosen to be presented here to focus on evolutionary traits rather than presumed ecological responses. The length of the flower pedicel varies from millimetres to a few centimetres long in the same species, while cataphylls that cover the primordium are assumed to be an adaptation to tolerance of drought or fire-prone environments (see ‘Discussion’ section for details). Development patterns analysed independently are available in Supplementary Figure S1. Character reconstruction models were selected from the default settings according to the best AIC weight through the function ‘fitMk’ of *phytools* and analysis of variance; these are ‘symmetrical’ for the branching pattern data set and ‘equal rates’ for reconstruction of the inflorescence patterns (AIC and computed values are provided in Supplementary Table S3, but see Supplementary Table S4 for alternative values for character reconstruction of seven development patterns and panicle). Stochastic mapping was performed using 10 000 simulations on both analyses. Transition rate scores from the analyses were used to build a matrix comparing transition rates among inflorescence patterns as input for a heatmap produced using the package *pheatmap* v1.0.12.

### Inference of time-calibrated phylogeny

The phylogenetic reconstruction of *Eugenia* used in this study was recovered using DNA sequences of the internal transcribed spacer (ITS: ITS1, 5.8S Gene, and ITS2) of the nuclear region (henceforward the ‘ITS dataset’) and four chloroplast regions (*psb*A*-trn*H*, rp*L*16, rp*L*32-trn*L*, trn*Q*-5’rps16*) (henceforward the ‘cpDNA dataset’). These sequences were acquired from previous molecular studies (Bünger *et al.* 2016a, Vasconcelos *et al.* 2017, Mazine *et al.* 2018, Giaretta et al. 2019a, 2019b). The analysis sampled 66 species (Table 1) selected as representative of the principal *Eugenia* lineages maximizing morphological and geographical diversity. Four taxa of tribe Myrteae were used as the outgroup to the phylogenetic reconstructions: two species of *Myrcianthes,* the sister genus to *Eugenia, Myrtus communis* and *M. tomentosa* (*sensu* Lucas *et al.* 2007).

Divergence times were estimated in BEAST v2.6.0 (Bouckaert *et al.* 2014) on XSEDE executed on the CIPRES portal (Miller *et al.* 2010). A monophyletic Eugeniinae (Lucas *et al.* 2019) was assumed and Birth–Death tree priors and an uncorrelated relaxed molecular clock model (general time reservable model and empirical base pair frequencies) were implemented on both nuclear and chloroplast partitions. Divergence times estimated for Myrteae microfossils were used as secondary calibration points following Vasconcelos *et al.* (2017) for the crown nodes of the *Eugenia* sect. *Umbellatae* (27.72 Mya) and Eugeniinae (29.29 Mya), implemented under normal priors and a standard deviation of 1.5. Four independent Markov Chain Monte Carlo runs, each of 50 million generations, were performed with a sampling frequency of 1000 trees. Outputs were examined using Tracer v1.7.1 (Drummond and Rambaut 2007) to assess chain convergence through verification of the effective sample size (ESS). Values of each parameter were considered satisfactory when ESS > 200. Results were combined in LogCombiner and a MCC tree selecting median height nodes was built using TreeAnnotator (both distributed as part of the BEAST package), discarding a burn-in of 10%. The resulting MCC time-calibrated tree was used as the phylogenetic foundation for the following traits reconstruction analysis.

## Results

### Main developmental patterns in the Eugeniinae inflorescence

The wide variety of arrangements in *Eugenia* rely on the flexibility of a few structures, i.e. (i) the extension and number of FSS arising from the same axil and their ultimate appearance; (ii) the branching potential of the bracteoles axil; and (iii) the potential to recover vegetative meristem activity at the FSS apex. The seven developmental patterns for *Eugenia* are described in Supplementary Table S5 and summarized below. A summary of the seven development patterns is provided. The three-flowered dichasium is treated as a secondary development and is here included under the *thyrsoid* development pattern.

**Figure 3:**
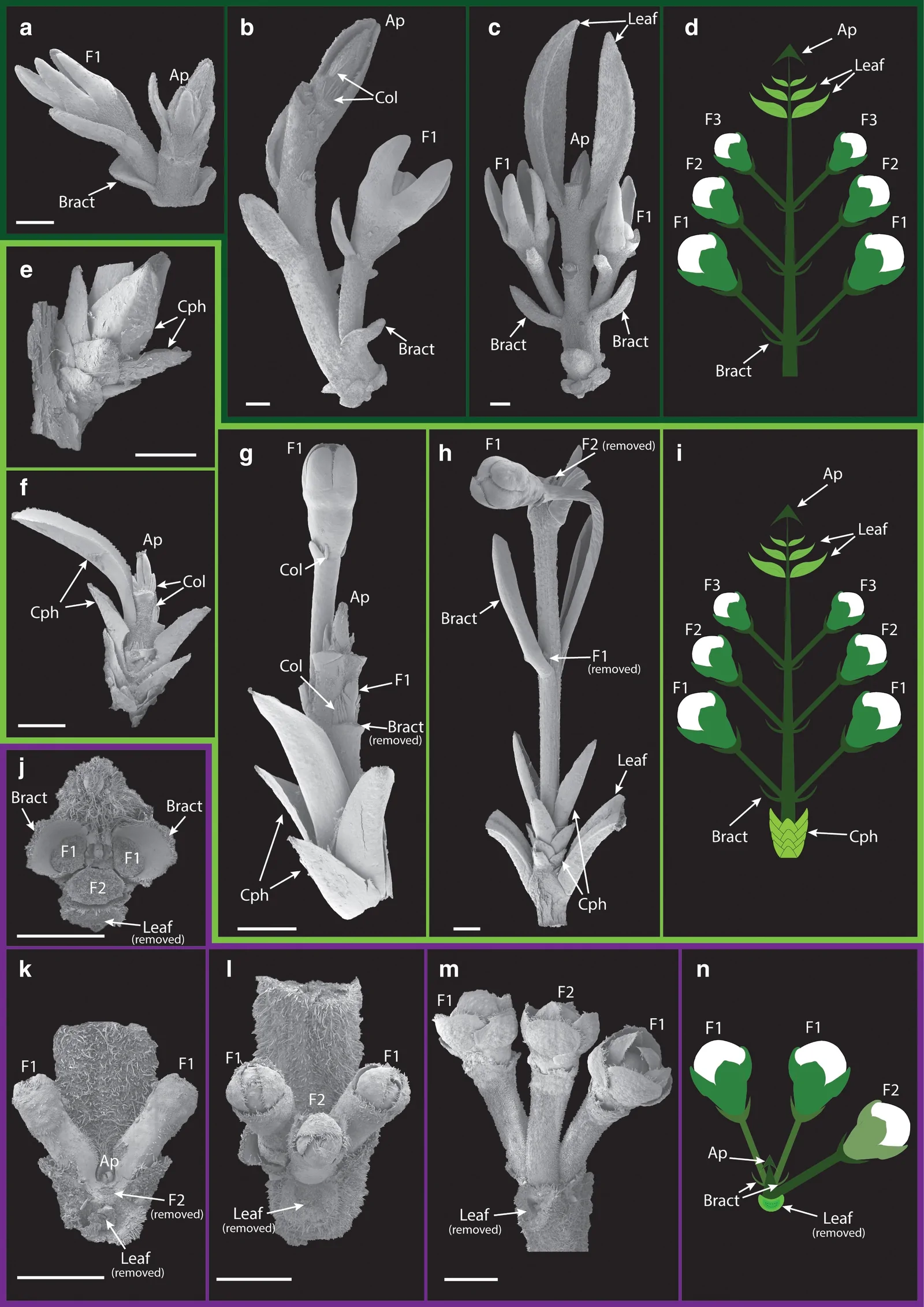
Comparison of inflorescence development patterns in *Eugenia*. (a–d—dark-green box) *Auxotelic* pattern: (a–c) Development of the flowering axis in *Eugenia bunchosiifolia*; (d) scheme representing the *auxotelic* pattern. (e–i—light-green box) *Auxotelic cataphylls*: (e) initiation of the flowering axis covered by cataphylls in *E. ligustrina*; (f) cataphylls are removed to reveal the primordium; (g–h) flowering axis development; (i) scheme of the *auxotelic cataphylls* pattern. (j–n—purple box) *Umbelliform* pattern: (j) upper view of the flowering axis of *E. sonderiana* with three flowers removed; (k–m) development of the flowering axis; (n) scheme representing the *umbelliform* pattern. Ap, apex; F1 to F3 represent flower pairs emerging in developmental sequence; Cph, cataphylls; Col, colleters. Scale bars = 1 mm.

**Figure 4:**
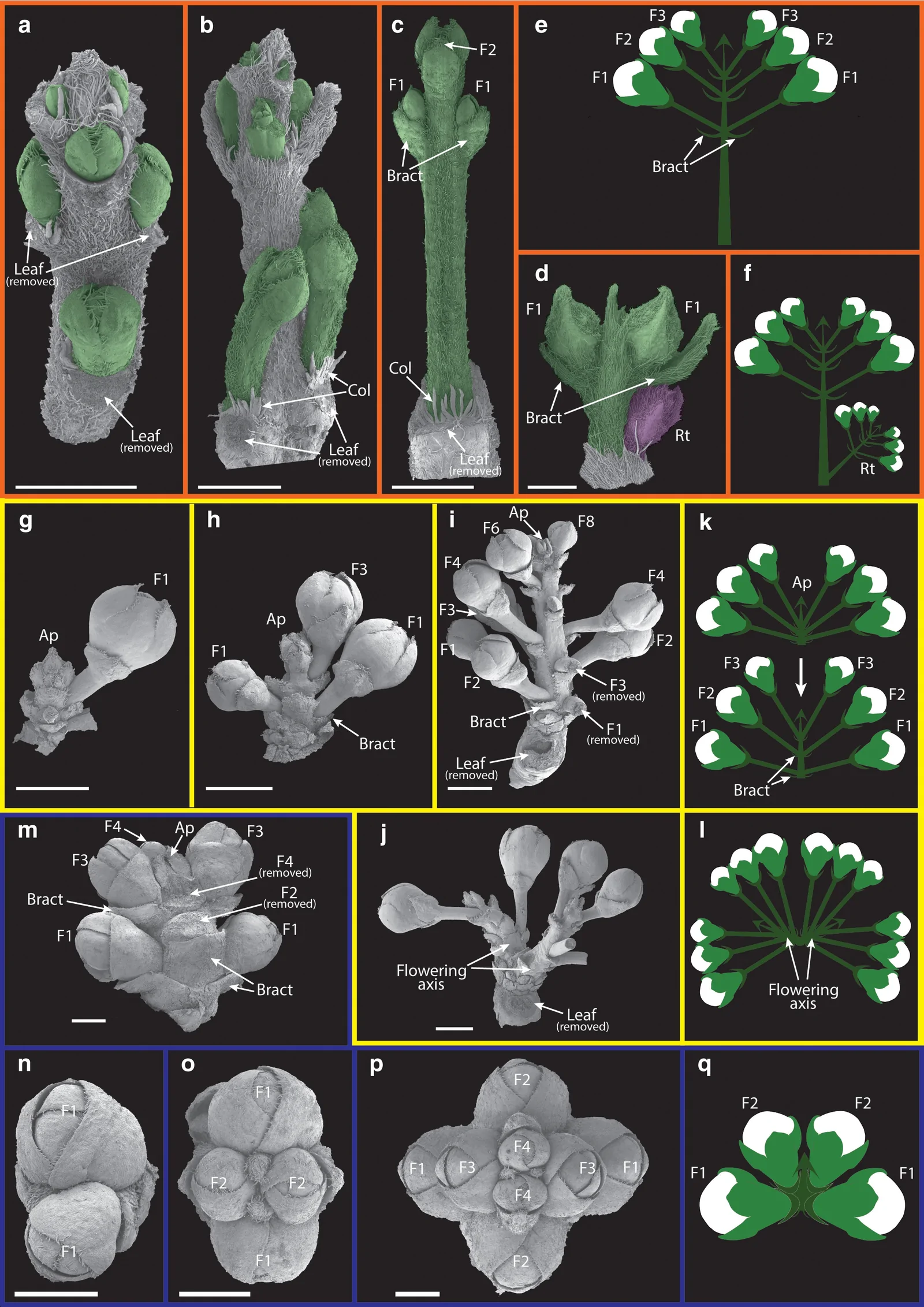
Comparison of inflorescence development patterns in *Eugenia*. (a–f—orange box) *Racemiform* pattern: (a–c) flowering axis (highlighted green) development before elongation of flower pedicels in *Eugenia modesta*; (d) delayed accessory flowering axis (highlighted purple) in *E. biflora*; (e) scheme representing the *racemiform* pattern; (f) scheme representing delayed accessory flowering axis. (g–l—yellow box) *Fasciculiform* pattern: (g–i) flowering axis development of *E. protenta*; (j) two flowering axes at the same development stage sharing an axil. (k) scheme representing the flexibility of the main axis length; (l) scheme showing two flowering axes at the same developmental stage sharing a common axil. (m–q—dark-blue box) Glomeruliform pattern: (m) lateral view of the flowering axis in *E. belemitana*; (n–p) flowering axis development from an upper view; (q) scheme showing the glomeruliform pattern. Ap, apex; F1 to F8 represent flower pairs emerging in developmental sequence; Col, colleters; Rt, flowering axis reiteration. Scale bars = 1 mm.

**Figure 5:**
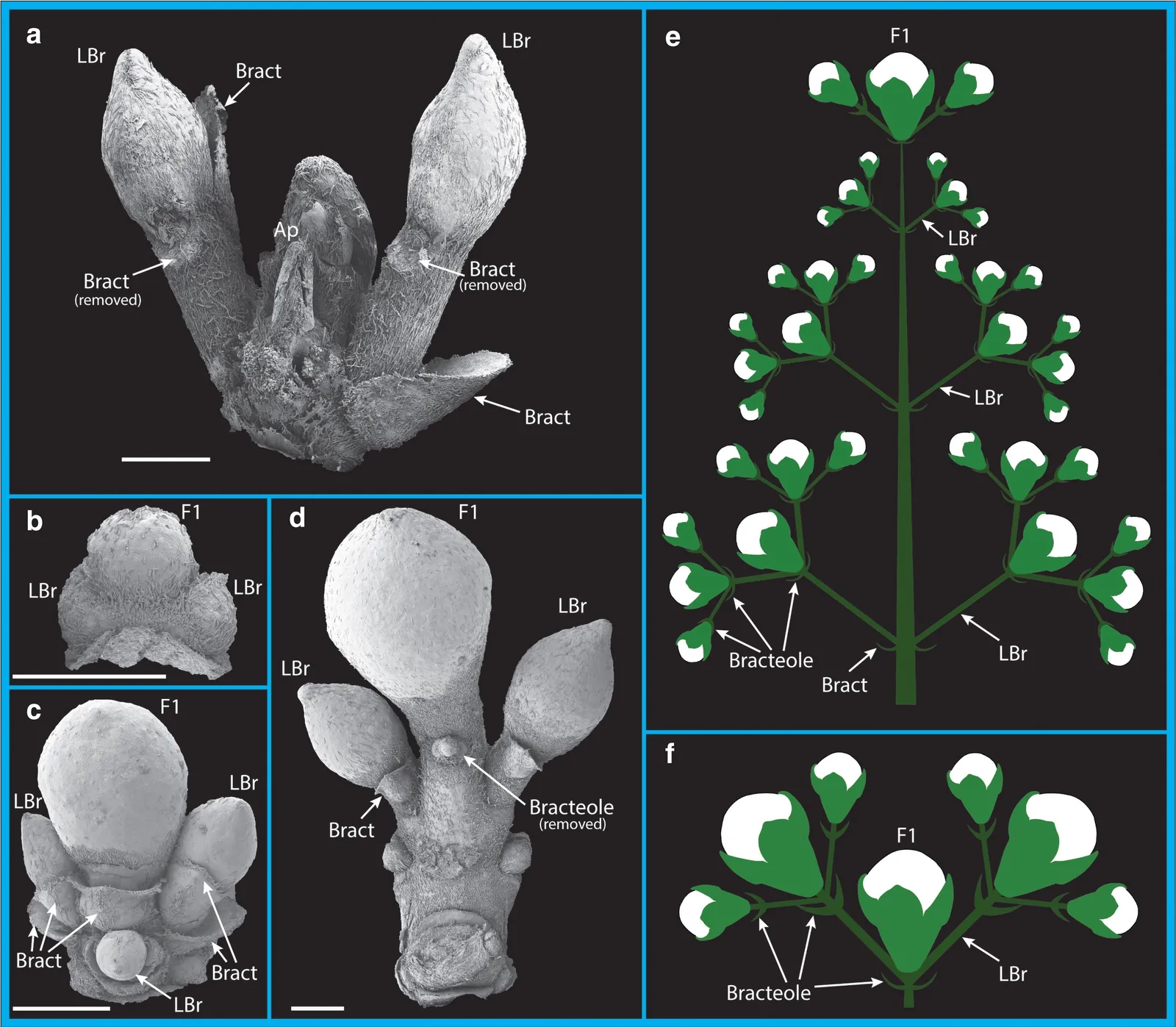
Comparison of inflorescence development patterns in *Eugenia*. (a–e—light-blue box) *Thyrsoid* pattern: (a) apical meristem retaining activity, resulting in early elongation of the lateral branching in *Eugenia guanabarina*; (b) early development of the *thyrsoid* pattern; (c) apical meristem overtaken by a central flower, potentially resulting in absent or minimal branching elongation; (d) central flower premature in relation to the lateral ones; (e) scheme representing the thyrsoid pattern. (f) scheme representing a dichasial arrangement. Ap, apex; F1 represents flower pairs emerging in developmental sequence; LBr, lateral branching. Scale bars = 1 mm.

The development of the FSS in *Eugenia* exhibits several distinct architectural patterns (Figs 3–5). In the *auxotelic* pattern (Fig. 3a–d), the main axis (first-order) bearing bracts subtending flowers arises from the axillary meristem of a previous season’s shoot (SPS), with lateral flowers (second order) developing acropetally from base to apex. The FSS initially may appear as a raceme with bracts, and the apical meristem remains active, maintaining vegetative growth that further extend. In the *auxotelic cataphylls* pattern (Fig. 3e–i), the FSS bears densely arranged cataphylls along short internodes; may appear as a raceme with bracts which distal flowers are subtended by leaves, and the apical meristem remains active that further extends. The *umbelliform* pattern (Fig. 3j–n) features a very short main axis subtended by a leaf and bearing 2–3 flowers (rarely 2–4 flowers) at tiny internodes, forming an umbel-like cluster with an inactive apical meristem. The *racemiform* pattern (Fig. 4a–f) shows a main axis subtended by a leaf whose first internode elongates before subsequent ones, supporting a compact group of second-order flowers; an accessory flowering axis with delayed development stage (in comparison to the main FSS) might be positioned between the leaf petiole and the main axis. In the *fasciculiform* pattern (Fig. 4g–l), the FSS, subtended by an often deciduous leaf, varies in length, producing either tight clusters or looser arrangements of flowers; multiple axes at the same development stage may share an axil, each ending in a vegetative meristem that does not further develop vegetative tissue. The *glomeruliform* pattern (Fig. 4m–q) involves a slowly elongating main axis with inconspicuous pedicels and apical primordia that does not further develop vegetative tissue, where two or more flowering axes at the same stage may share an axil. Finally, in the *thyrsoid* pattern (Fig. 5a–f), the main axis with bracts bears acropetally arranged lateral branches or flowers; each flower is subtended by paired bracteoles with further branching potential, forming a pyramidal structure that terminates in a sessile flower and a ceased vegetative meristem.

### Phylogenetic relationship and ancestral reconstruction

The dated phylogeny recovered all 11 infrageneric groups to provide a coherent framework for the reconstruction of the inflorescence development patterns here described. The backbone of *Eugenia* has good support in the combined dataset, with ca. 80% of the nodes with ≥0.95 posterior probability. Internal relationships are also strong, with c. 70% of the nodes with >0.95 PP. Divergence times of the stem and crown nodes of *Eugenia* were 29.6 Mya (27–32.2 Mya) and 26.6 Mya (23.3–29.8 Mya), respectively.

**Figure 6:**
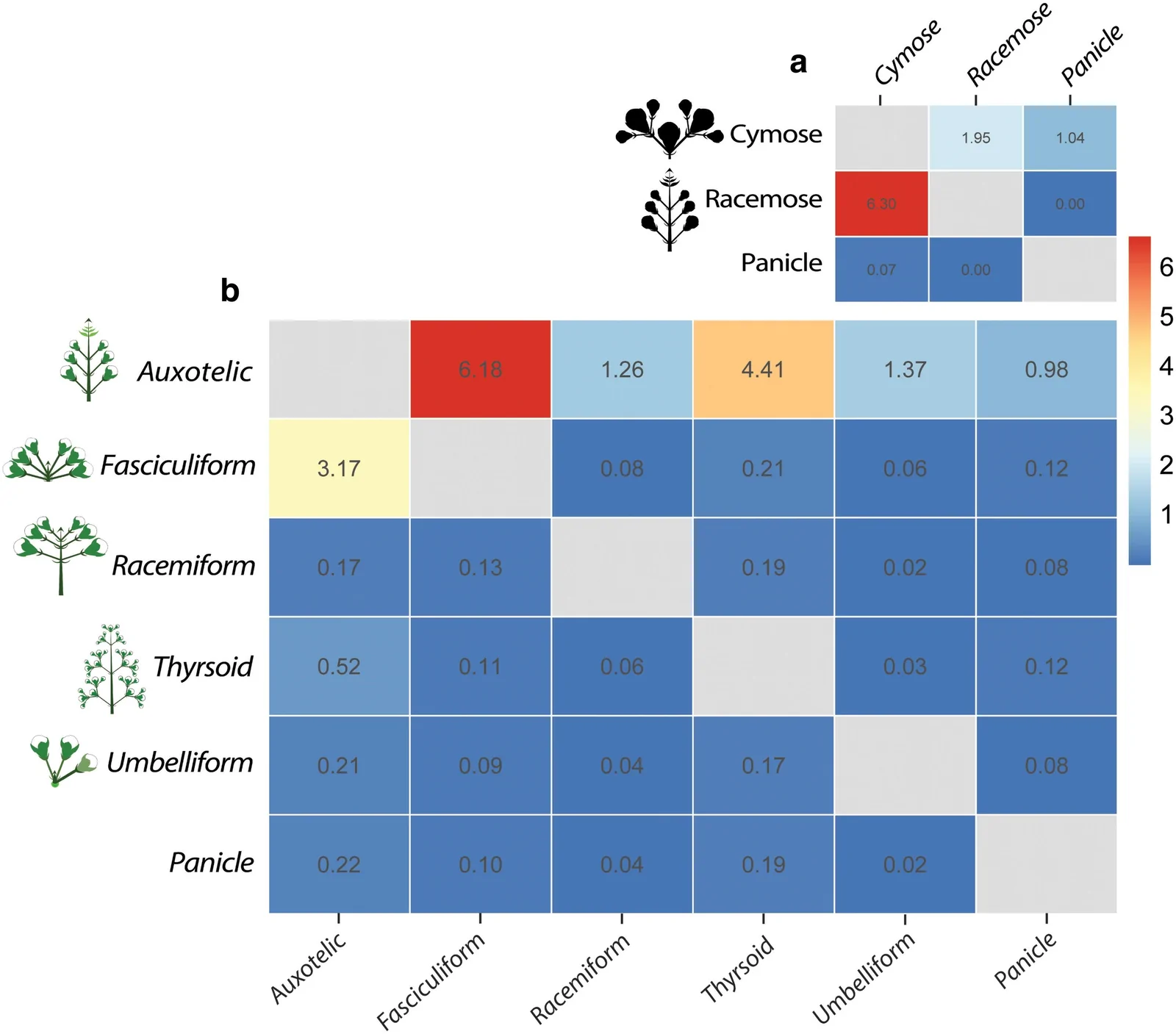
Heatmap of transition rates between inflorescence patterns in *Eugenia*. (a) Shows transition rates of inflorescence patterns according to their branching patterns; (b) displays transitions among the five inflorescence patterns and panicle indicated. The heatmap is read from left to right, with each cell representing the mean number of transitions from the pattern in the row to the pattern in the column across 10 000 stochastic maps; for example, transitions from *auxotelic* to *fasciculiform* were estimated to occur 6.18 times on average, whereas reversals from *fasciculiform* to *auxotelic* occurred 3.17 times.

Transition rates of branching patterns depicted in the heatmap suggest that the inflorescence of *Eugenia* changed approximately nine times between states on average with most transitions from racemose to cymose (6.3 times), while reversions (1.9) and transitions from cymose to panicle (1.0) are less prone to occur (Fig. 6a). Panicle exclusively found in the outgroup represented by *M. tomentosa* recorded low-rate transitions from or to the cymose pattern, while switches involving the racemose pattern do not occur. The five inflorescence patterns had 20.5 transitions between states on average. Transitions from the *auxotelic* to any other pattern are associated with an increase in the transition rates, especially to the *fasciculiform* and *thyrsoid* patterns that scored 6.1 and 4.4 transitions, respectively (Fig. 6b). In contrast, reversions to the *auxotelic* pattern are less inclined to occur, with scores ranging from 0.1 to 0.5 transitions on average except for transitions to the *fasciculiform* pattern that scored 3.1 transitions on average.

**Figure 7:**
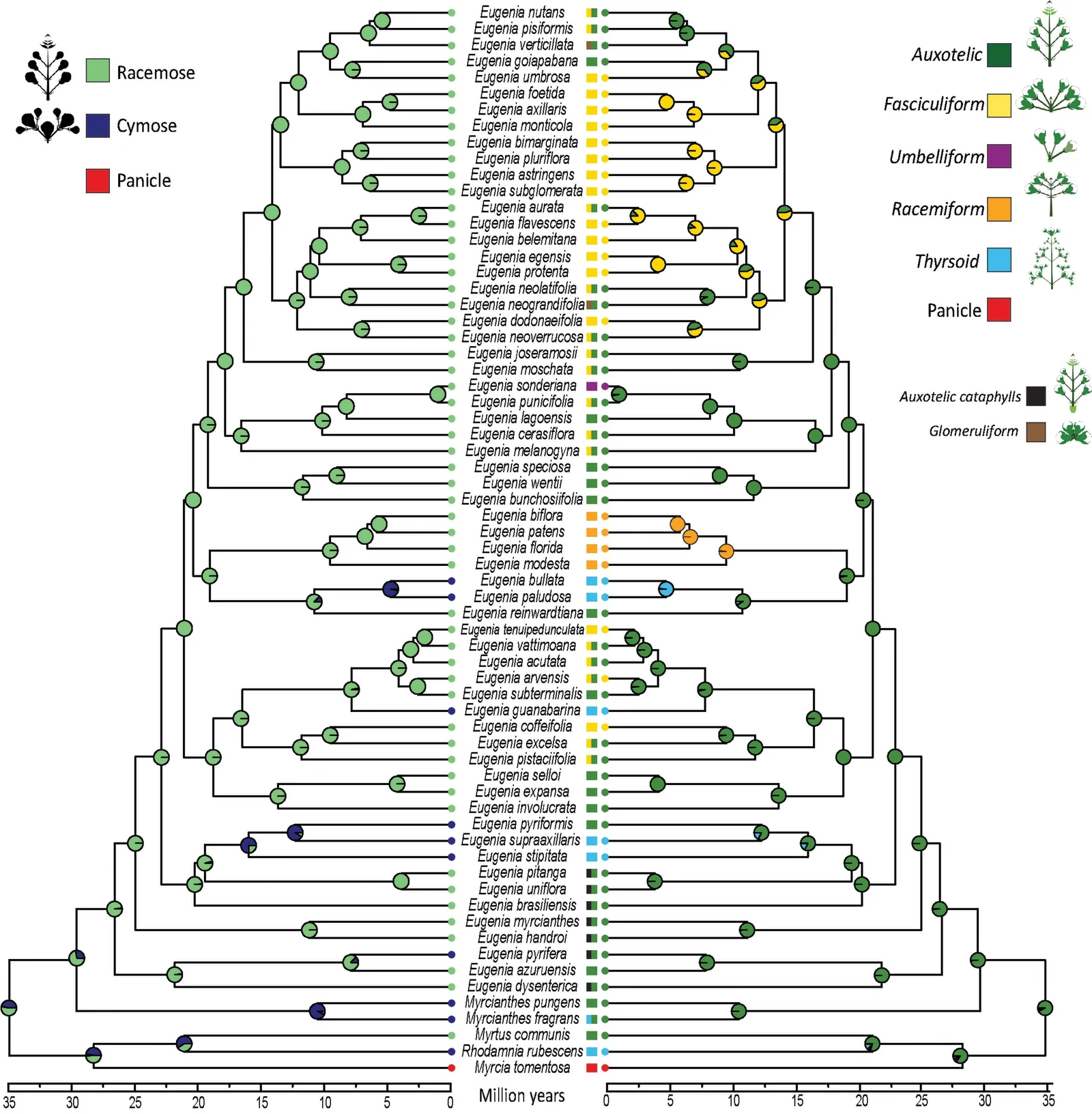
Time-calibrated phylogeny of *Eugenia* with ancestral nodes of inflorescence development patterns inferred from 10 000 stochastic character maps. Ancestral branching pattern probabilities are shown as pie charts at nodes on the left-hand tree; note that branching in *Eugenia* is primarily racemose, and lineages expressing secondary cymose architecture are scored accordingly. Ancestral inflorescence pattern probabilities are shown as pie charts at nodes on the right-hand tree. Coloured rectangles at the tips indicate the observed development patterns for each species, whereas coloured circles indicate the states coded in ancestral character reconstruction analysis. *Auxotelic cataphylls* and *glomeruliform* were distinguished only at the tip level to code the observed variation (rectangles) but were pooled with *auxotelic* and *fasciculiform* patterns, respectively, for the reconstruction analyses to focus on broader evolutionary patterns rather than fine-scale ecological responses.

Character reconstruction of the branching patterns recovered a recurrent expression of the cymose pattern associated with lineages originating at earlier diverging nodes. The racemose pattern was more frequently associated with lineages from later diverging nodes (Fig. 7). Reconstructions of the inflorescence patterns showed that the *auxotelic* pattern is more likely to be the ancestral condition of the node shared by *Eugenia* and *Myrcianthes* and the most probable ancestral condition at all nodes until the diversification within *Eugenia* sect. *Umbellatae*. *Auxotelic* and *auxotelic cataphylls* were scored as a single state in the character reconstruction (see Material and Methods—but see Supplementary Figure S1 for alternative reconstruction analysis with seven development patterns and panicle). The *umbelliform* pattern is found to be the most likely state at earlier diverging nodes of *Eugenia* sect. *Umbellatae*, represented here only by *Eugenia sonderiana*. *Fasciculiform* and *glomeruliform* patterns were also scored as a single state and appear to be a feature in the later evolutionary history of *Eugenia*. Those latter patterns are exclusive to *Eugenia* sect. *Umbellatae*, except for *E. coffeifolia* (assigned as *glomeruliform*) that emerges in *Eugenia* sect. *Excelsae*. The *racemiform* pattern appears to have arisen only once and is exclusive to *Eugenia* sect. *Racemosae*. The *thyrsoid* pattern apparently evolved independently at least four times within *Eugenia* and is more common in lineages originating at earlier diverging nodes. The reconstructed ancestral node of the extra-neotropical lineage appears most likely to have been *auxotelic*.

**Figure 8:**
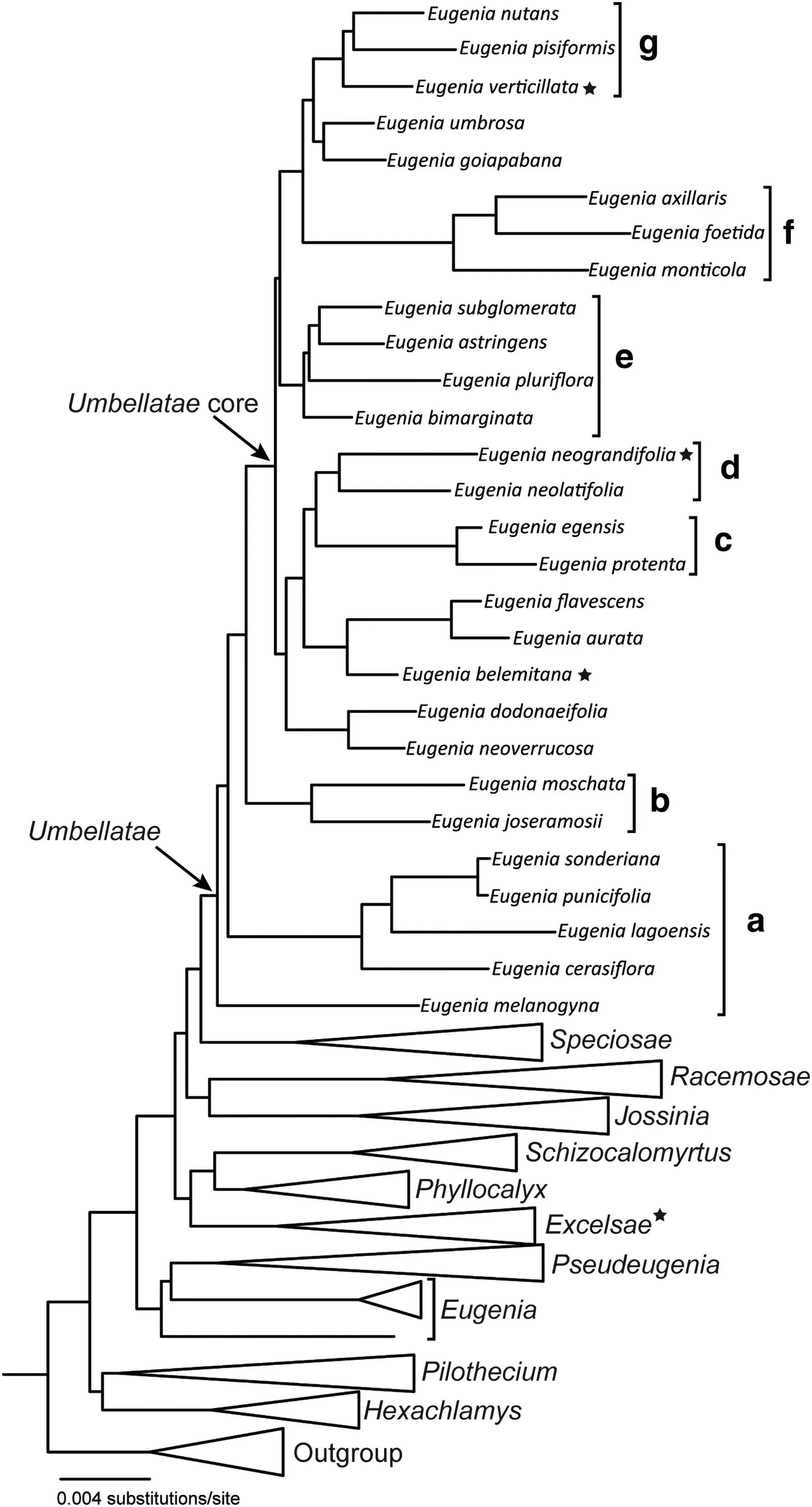
Phylogenetic tree summarizing *Eugenia* with a focus on *Eugenia* sect. *Umbellatae*. Lettered clades correspond to discussion in the text. ★Signifies species that fit the *glomeruliform* pattern.

Traits associated with clades were identified as relevant to diagnose subgroups within *Eugenia* sect. *Umbellatae*. The condition where two or more flowering axes develop simultaneously, sharing an axil that never recovers vegetative growth, is consistently associated with the *fasciculiform* pattern recurrently emerging in clades C, E, and F (Fig. 8). Conversely, the condition of a single flowering axis that often recovers the vegetative growth is associated with the *auxotelic* pattern emerging in clades A, D, and G. The only exception was observed in clade B where the ancestral node state was assigned as the *auxotelic* pattern but where the inflorescence often expresses at least two flowering axes in a shared axil.

## Discussion

### Inflorescence architectures

Whether the inflorescence is determinate or indeterminate is not relevant to assign branching pattern according to Endress (2010). Instead, classification relies on hierarchical branching number, i.e. number of branching orders. The number of branching orders is limited to two in the racemose branching pattern, i.e. the main axis (first-order) is fixed, whereas the number of branching axes (second-order) corresponding to flowers is variable and unlimited, except for compound racemes with repetition of a basic arrangement. In a cymose branching pattern, the number of branching orders is unlimited, i.e. the number of lateral branches is limited to two, but the number of orders is variable (Endress 2010). These basic arrangements can repeat successively resulting in more complex scaffoldings.

### Plant architectural traits relative to inflorescence interpretation

Architecture is a consequence of how flower and inflorescence traits are arranged relative to each other, their relative position, and successive repetition. As a result, plants are modular organisms of multidimensional processes driven by endogenous growth and exogenous constraints that define their form and resulting architecture (Barthélémy and Caraglio 2007, Chomicki *et al.* 2017). Plant architectural traits must be understood before considering general inflorescence development.

Repetition of partial or whole architectural units from otherwise dormant meristems has been termed delayed reiteration (Chomicki *et al.* 2017) and is clearly implicated in the architectural expression of neotropical *Eugenia* and Myrteae. The *auxotelic* pattern is here treated as a reiterated SSS with bracteose or foliose leaves but when subtending axillary flowers are presented, it is treated as an FSS. *Auxotelic* reiterations produce more axils that give rise to axillary reproductive units, increase branching order in plant’s architecture, and ultimately contribute to vegetative production allowing exploitation of space. Apical meristems can also differentiate into reproductive units and express inflorescence arrangements that reflect architecture nature of the plant. Meristematic buds are maintained at the apex of branches or inflorescence units termed indeterminate (Troll and Weberling 1989, Weberling 1990) or blastotelic (Briggs and Johnson 1979). Two types of indeterminate behaviours are seen in the tribe, i.e. (i) a pattern that ceases growth at some stage, often early in the development, and (ii) a vegetative meristem that ceases growth but later recovers the capacity to grow and gives rise to a shoot (late proliferation). Overall, in plants, apical dominance presumably moderates inflorescence branching (Harder and Prusinkiewicz 2013) and is related to the meristematic vigour with which vegetative tissue is produced (Claßen-Bockhoff and Bull-Hereñu 2013).

### Phylogenetic implications of branching patterns

Racemose and cymose branching patterns are recognized as fundamental inflorescence architectures (Endress 2010); both are found in *Eugenia*. The racemose branching pattern in Myrteae is here assumed to be the ancestral condition for *Eugenia* (Fig. 7). This is supported by the high likelihood of the racemose pattern recovered as the ancestral states at the majority of nodes of the backbone (Fig. 7) and higher transition rates from racemose to cymose (Fig. 6). The cymose inflorescence is the standard pattern in *Eugenia*’s sister genus, *Myrcianthes*. The cymose pattern appears secondary in *Eugenia,* apparently derived from a primary racemose pattern. Vigour in branching potential from bracteole axil modulates a cymose arrangement. Therefore, cymose branching is always partial in *Eugenia* as it is a derivation of a racemose primary architecture. This is the case for *Eugenia* sect. *Pilothecium* and sect. *Pseudeugenia* that have a conserved racemose branching but with recurrent dichasial arrangements emerging from bracteole axils (see Faria 2014).

### Homologous arrangements in light of current evolutionary understanding

*Myrtus*, sister to the Neotropical lineage, and *Myrcianthes*, sister to *Eugenia*, express the *auxotelic* development pattern that are the most probable ancestral state at the earliest nodes of the *Eugenia* phylogeny originated around 27 Mya (Fig. 7) as well as indicating the highest transition rates to any other development pattern (Fig. 6). Thus, it is hypothesized here that the ancestral inflorescence arrangement of *Eugenia* is similar to the *auxotelic* pattern, hereafter referred to as the hypothetical auxotelic ancestral (HAA) pattern. This suggests that flexible arrangements found in *Eugenia* are a result of plastic phenotypes of the basic HAA arrangement, and also, that evolutionary transitions between development patterns belong to a spectrum of continuities rather than being purely homoplastic conditions. The concept of the HAA supports the view of McVaugh (1956), who suggested that the inflorescence architectures of Myrtaceae are derived from a single basic arrangement.

The conservative occurrence of the auxotelic arrangement across multiple lineages (see *Auxotelic* pattern in Fig. 7) may be related to the efficiency of this architecture, allowing synchronized phenophases and ultimately saving energy, supporting both photosynthetic leaves and reproductive units. In contrast, species with a *fasciculiform* pattern, which is widespread in *E*. sect. *Umbellatae*, bear flowers emerging from axils along the branches as a strategy to increase floral display. As a consequence, meristems are committed to reproduction. Inflorescences that retain meristems capable of later recovering vegetative growth in an auxotelic fashion are therefore favoured (Fig. 7). The auxotelic arrangement is indeterminate, as all other patterns in *Eugenia* except the thyrsoid (see ‘Capacity to recover vegetative growth’ in Table 2). However, there is a tendency in *Eugenia* for inflorescences to be functionally determinate: *umbelliform*, *racemiform,* and *fasciculiform* patterns often show growth that is repeatedly terminated by a vegetative bud. Determinate inflorescences are associated with more synchronous flowering over short periods, which can optimize pollination under favourable conditions (Stebbins 1973, Shao et al. 2026), in agreement with the mass flowering of Neotropical Myrtaceae typically in spring and early summer (Proença and Gibbs 1994). A biogeographic assessment of inflorescence types showed that lineages with determinate inflorescences are concentrated in tropical environments and exhibit significantly higher speciation rates than lineages with indeterminate inflorescences (Wang et al. 2025). Functionally determinate inflorescences arose several times in *Eugenia* but significantly thrived around 19 Mya in the clade including *E*. sects. *Racemosae* and *Jossinia*, and around 16 Mya in *E*. sect. *Umbellatae* (Fig. 7). The *fasciculiform* inflorescence that recovers its vegetative growth is widespread in *E*. sect. *Umbellatae*, which harbours half of the genus diversity and has the broadest distribution, including long-distance dispersal to the Caribbean (Mazine et al. 2018, Flickinger et al. 2020). This combination of intense flower display observed in the *fasciculiform* pattern and vegetative flexibility may represent a key innovation that has enabled *E*. sect. *Umbellatae* adapt to contrasting climatic conditions and generate species diversity.

**Table 2:** Comparative traits of inflorescence development patterns.

Proposed terminology	Auxotelic		Umbelliform	Raceme	Fasciculiform		Thyrsoid
Development patterns	*Auxotelic*	*Auxotelic cataphylls*	*Umbelliform*	*Racemiform*	*Fasciculiform*	*Glomeruliform*	*Thyrsoid*
Branching pattern	Racemose	Racemose	Racemose	Racemose	Racemose	racemose	Racemose and cymose
Subtending structure of the flower	Foliose	Foliose	One foliose (shared with the main axis) and others bracteose	Bracteose	Bracteose	Bracteose	Bracteose
Cataphylls densely arranged	Absent	Present	Absent	Absent	Absent	Absent	Absent
Persistence of the main axis bract/leaf	Persistent	Persistent	Persistent	Persistent	Usually deciduous	Usually deciduous	Persistent
Main-axis relative length	Long	Long	Very short	Moderate	Short or moderate	Short	Long
Frequency of dichasial arrangement expression	Low	Moderate	Not observed	Very low	Low	Not observed	High
Main-axis internodes extension	Homogeneous	Heterogeneous	Homogeneous	Heterogeneous	Homogeneous	Homogeneous	Homogeneous
Pedicel extension in the flower development	Early	Early	Early	Late	Early	Inconspicuous	Early
Capacity to recover vegetative growth	Present	Present	Ceased by vegetative a bud	Ceased by vegetative a bud	Ceased by vegetative a bud	Ceased by vegetative a bud	Ceased by a flower
Number of main axes sharing an axil and relative position	One	One	One	Two (rarely three) aligned	Up to three in arbitrary position	Up to three in arbitrary position	One
Reiterated flowering axis	Not observed	Not observed	Not observed	Delayed	At the same development stage	At the same development stage	Not observed

### Homologous transitions among inflorescence patterns and ecological significance

The developmental sequence of *auxotelic cataphylls* is essentially *auxotelic* except for a basal zone of condensed cataphylls that cover the primordium. The protected bud is an adaptation to tolerance of fire-prone environments (Charles-Dominique *et al.* 2015). Lineages expressing the *auxotelic cataphylls* pattern first originated around 25 Mya, coinciding with the origin of endemic lineages associated with the Brazilian savanna Cerrado from the late Miocene onward (Simon *et al.* 2009). The ancestral habitat of *Eugenia* has been suggested to be in forests associated with drier biomes (Mazine *et al.* 2018), where many species with the *auxotelic cataphylls* pattern currently occur. Lineages from early diverging nodes in *Eugenia* and in other genera, as well as *Eugenia excelsa* and *E. pistaciifolia*, occur in warmer, more drought-prone environments (see Souza *et al.* 2008, Faria 2014, Stadnik *et al.* 2018). This suggests that cataphylls may be related to protection against dehydration or fire.

Results suggest that lineages with a *fasciculiform* pattern first originated around 16 Mya in a clade shared with sects. *Schizocalomyrtus* and *Excelsae*, and independently in *E*. sect. *Umbellatae* (Fig. 7). Apparent reversions from the *fasciculiform* to the *auxotelic* pattern were recorded in *Eugenia* sect. *Umbellatae* and in other sections of *Eugenia* (e.g. sect. *Hexachlamys*, see *E. handroi*) with relevant transition rates (Fig. 6). Flowering axis length reduction is common in *E.* sect. *Umbellatae*. However, the apical meristem eventually recovers its vegetative activity in some species and develops into a shoot. This condition is recurrent in some species, consistent with their clades (Fig. 8, see nodes A, D, and G) and is scored as *auxotelic* rather than *fasciculiform*. Shifts in the main axis length and expression of the *auxotelic* pattern may influence the floral display and enhance space exploitation. The inflorescence architecture was demonstrated to influence the pollinator behaviour (Harder *et al.* 2004, Ishii *et al.* 2008), while fruit position through the inflorescence has an impact on its size (Wyatt 1982, Torices and Méndez 2010).

The *umbelliform* pattern in *Eugenia* was recorded only once, represented by *E. sonderiana*. The arrangement of the subtending leaf simultaneously shared with the flowering axis and a flower is unique in *Eugenia* and has not been noticed before as far as we know. Although this arrangement makes the *umbelliform* pattern easily distinguishable, suppression of structures in developmental sequence decreases the accuracy of the architectural inference (Prenner *et al.* 2009). Despite this, the flowering axis of the *umbelliform* pattern is here treated as homologous to the main axis of the HAA. This is inferred by the slightly advanced developmental stage of the F1 flower pair in contrast to F2, reinforced by the consistent decussate developmental arrangement observed and no reiteration recorded (Fig. 3j and m). Availability of space has an important role in the development of *Eugenia* (Vasconcelos *et al.* 2018) and is highlighted as the main driver of the recurrent three-flowered arrangement observed in the *umbelliform* pattern. This hypothesis is supported by the occasional four-flowered arrangement expressed at the terminal node of *E. sonderiana*, presumably in response to greater available spatial resource. Suppression of the bract that subtends F2 is seemingly a consequence of the spatial constraint resulting from the very short main axis.

Developmental sequence and final arrangement provide a consistent framework to distinguish the *racemiform* pattern. The flower of the HAA is interpreted as homologous to the flowering axis in the *racemiform* pattern. This means that the FSS of a *racemiform* inflorescence (see Fig. 2) corresponds to the axillary flowering axis subtended by leaves from the shoot of the current season. This view is supported by the apex of the FSS that never recovers its potential for growth. In addition, a delayed accessory flowering axis found in the *racemiform* pattern is a similar condition found in *M. communis*, where instead of a flowering axis, a delayed accessory flower positioned between the leaf and the main flower is recurrent. This is here considered as a plesiomorphic mechanism to increase flower availability to pollinators and is presumably distinctive to the superimposed arrangement found in *Myrceugenia*, where a sequential row of accessory units in different development stages is expressed in a single leaf axil (e.g. Landrum and Kawasaki 1997).

*Fasciculiform* and *glomeruliform* inflorescences have identical developmental patterns, except for the absence of a pedicel in the latter arrangement which is likely related to stochastic factors (see further discussion in the topic "Assessement of traditional terminology"). As a result, we here treat both patterns as *fasciculiform*. The flower of the HAA is interpreted as homologous to the flower of the *fasciculiform* pattern. Therefore, the FSS of the *fasciculiform* pattern (Fig. 2) is homologous to the flowering axis of the HAA. This is supported by a main axis of the *fasciculiform* inflorescence subtended by a bracteose or foliose structure of variable length ranging from millimetres to a few centimetres long observed even in the same species (see *Eugenia itaunensis* Giaretta & Peixoto, Giaretta *et al.* 2018). Alternatively, a *fasciculiform* structure retains unique features such as a vegetative meristem at the apex that has ceased growth, therefore, unable to recover vegetative development. The most remarkable feature of the *fasciculiform* pattern is the capacity for two or more flowering axes at the same development stage to share an axil. This is here treated as reiteration of the FSS and is diagnostic of this pattern. Thus, this reiteration, and delayed accessory flower/axis, exclusively observed in the *racemiform* pattern, are interpreted as analogous strategies for increasing the number of available flowers. The ‘*Eugenia moschata* group’ does not follow this pattern (Fig. 7—clade b) as discussed below.

The switch from the *auxotelic* to the *thyrsoid* development pattern is associated with increased transition rates (Fig. 6) as a consequence of a shared primary racemose arrangement. This supports the hypothesis that second-order branching of the *thyrsoid* inflorescence is homologous to a flower of the HAA, and that further orders result from vigorous branching potential expressed as successive subtending bracteoles. Although the main axis is terminated by a flower, the determinacy of the apex is not relevant to the assigned branching pattern (Endress 2010). This is reinforced by the enormous numbers of parallel origins of the terminal flower among angiosperms (Bull-Hereñu and Claßen-Bockhoff 2011).

### Systematic implication of the inflorescence patterns

The *auxotelic* pattern is commonly found in infrageneric groups such as *Eugenia* sect. *Phyllocalyx* and sect. *Speciosae* (Fig. 7). However, this pattern also occurs in other lineages of *Eugenia* as demonstrated by recurrent transitions from *auxotelic* to any pattern (Fig. 6). A combination of additional morphological characters should be used to consistently diagnose these sections (Bünger *et al.* 2016a, 2016b). The *auxotelic cataphylls* pattern was recovered independently at least seven times (see Supplementary Figure S1) in earlier diverging lineages such as *Eugenia* sect. *Hexachlamys*, sect. *Pilothecium*, sect. *Eugenia,* and sect. *Pseudeugenia*. These sections have significant diversity in the Brazilian *Cerrado* (Mazine *et al.* 2016, 2018), a warmer and dryer environment compared to Atlantic Forest, suggesting a possible correlation between the *auxotelic cataphylls* pattern and the environment.

*Racemiform* pattern originated around 9.5 Mya, coinciding with the diversification of *Eugenia* sect. *Racemosae* (Mazine et al. 2018). The *racemiform* pattern is consistently associated with *Eugenia* sect. *Racemosae* and has been defined based on the relative length ratio between the pedicel and flower internodes (Mazine *et al.* 2016). Additionally, an accessory flowering axis with delayed development (in contrast to the main axis) is exclusive to this section and can be consistently used for infrageneric diagnosis. *Eugenia* sect. *Jossinia* is represented by a thyrsoid inflorescence, with material studies demonstrating a combination of racemose and cymose arrangements in contrast to the racemose arrangements mostly found in neotropical species (Fig. 7). Inflorescence development studies focusing on the extra-neotropical species may indicate specific arrangements as cymose influencing migration events to other regions.

The relative length and number of the main axes that arise from an axil are shown to be systematically relevant to recognize clades within *Eugenia* sect. *Umbellatae*. The *fasciculiform* pattern assessed with the *glomeruliform* pattern is most commonly recovered in clades C, E, and F (Fig. 8). Additionally, in this pattern, the main axis does not develop vegetative tissue beyond the flowering region. Conversely, an independent flowering axis that morphologically resembles *fasciculiform* but never shares an axil with a flowering axis and often recovers the vegetative growth is coded as the *auxotelic* pattern recovered in clades A, D, and G (Fig. 8). The only exception within *E*. sect. *Umbellatae* is when an axil simultaneously bears two or more flowering axes at the same development stage that then can recover the vegetative growth, as in the ‘*Eugenia moschata* group’. This combination of features can help to diagnose this clade (Fig. 8, clade b).

### Assessment of traditional terminology

This is the first attempt to integrate our current knowledge of the evolution of different inflorescence architectures in *Eugenia* within a single circumscription. The resulting system accounts for initial development as well as the flexible arrangements of mature inflorescences, creating more comparative and unified terminology. Terminology traditionally used in Myrtaceae relies on the definition of the most common arrangements in a given taxon based on mature material (e.g. Landrum and Kawasaki 1997), treating flexibility in arrangement as exceptional (see Briggs and Johnson 1979). These ‘exceptions’ increase the possibilities of phenotype and hamper accuracy in applying terminology. Rather than seeking further definitions of arrangements or newly introducing terms, the results of this work improve application of the existing terminology.

The definition of the ‘auxotelic conflorescence’ by Briggs and Johnson (1979) relies on the capacity of the apex to recover vegetative growth while axillary flowers (or flowering axes) emerge. The *auxotelic* and *auxotelic cataphylls* patterns are here described in greater detail, expanding the circumscription of the term auxotelic (see Table 2 and Supplementary Table S5). The *auxoletic/auxotelic cataphylls* pattern is equivalent to the system of solitary flowers described by Landrum and Kawasaki (1997) in a broader perspective, a ‘proliferating synflorescence’ (Weberling 1990) or ‘Stenocalyx type’ (McVaugh 1968, Barroso *et al.* 1991). Sometimes, the main axis expresses late proliferation by ceasing growth and later recovers the capacity to growth (see Prenner *et al.* 2009). Therefore, the auxotelic inflorescence can be expressed early in the development of some species, as equivalent to the ‘solitary flower’ (*sensu* Landrum and Kawasaki 1997), or when it later recovers vegetative activity from a fasciculiform flowering axis. Assignment of an auxotelic inflorescence should be followed by an annotation of both these inflorescence arrangements in species where they simultaneously occur. Although the focus of the terminological assessment is on the genus *Eugenia*, this expanded terminology for the auxotelic arrangement can be applied to other Myrtaceae.

The *racemiform* pattern is here associated with the term raceme in order to best circumscribe its use. Raceme is suggested to be adopted in reference to *Eugenia* sect. *Racemosae* (*sensu* Mazine *et al.* 2016) since it can be consistently recognized based on the development and mature morphology as well as to arrangements that fit to the *racemiform* pattern here described. This term has been used to describe inflorescence arrangements of species from *E*. sect. *Racemosae* (Souza and Morim 2008, Mazine and Souza 2010).

The term ‘fascicle’ is used inconsistently in taxonomic literature specific to *Eugenia*, commonly referring to an axillary flowering axis with a short main axis (e.g. Lourenço *et al.* 2013, Barrie *et al.* 2016). The length fluctuation of the main axis encourages the use of terms such as raceme, short-raceme (Souza *et al.* 2015), fasciculiform (Sobral *et al.* 2012) or fasciculate (Barrie *et al.* 2016), adding more confusion to the plethora of terminologies. Thus, fasciculiform term is here suggested to be adopted in allusion to the *fasciculiform* and *glomeruliform* patterns. Fasciculiform rather than fascicle should be used due to the flexible length of the main axis of the former. This is in contrast with the typological definition of the fascicle (e.g. Weberling 1988) as a raceme with a very short main axis that does not entirely fit into the *Eugenia* morphological span. Although the absence of pedicel impacts the ultimate fasciculiform floral display, results presented here suggest variation of this nature can be more related to stochastic factors affecting populations than inheritance. The fasciculiform arrangement in nature can simultaneously express both sessile and pedicellate flowers (e.g. see *E. neoglomerata—*currently *E. subglomerata*—in Lima *et al.* 2015). Fasciculiform patterns where flowers are sessile often display two or more flowering axes (at the same development stage) sharing a node that never recovers the vegetative growth (see *Eugenia subglomerata*, Fig. 8, clade e). Alternatively, species with sessile flowers that exclusively display a single flowering axis per node and commonly recover late vegetative growth fit the *auxotelic* pattern (see *Eugenia verticillata*, Fig. 8, clade g).

Umbelliform and umbel are recurrent in the taxonomic literature (e.g. Sobral *et al.* 2012, Faria *et al.* 2015) but are mostly related to *fasciculiform* arrangements with a very short main axis. The typological definition of umbel refers to a raceme with a compressed main axis (Weberling 1990), however, this is a generalist definition that cannot be accurately applied to *Eugenia*. Thus, it is here suggested that the term umbelliform should be referred to as the *umbelliform* pattern, where a very short main axis expresses a recurrent three-flowered arrangement. This pattern is apparently rare in *Eugenia*.

Thyrsoid, first employed by Briggs and Johnson (1979), has been used in the taxonomic literature in a conservative sense. Although the thyrsoid arrangement is flexible, its recognition is facilitated by the unique combination of the racemose branching pattern expressed in the first-order and cymose branching in further orders (Endress 2010). Metabotryoid (*sensu* Briggs and Johnson 1979) is treated as flexibility in the thyrsoid, an arrangement where the branching potential of the axil subtended by the bracteole ceases. We here suggest this term should be referred to as the *thyrsoid* pattern in neotropical *Eugenia*.

The dichasium is often referred to as an inflorescence in the Myrtaceae literature (McVaugh 1969, Faria *et al.* 2015). However, the dichasial arrangement is here recognized as a secondary architecture in the evolution of *Eugenia,* where the ancestral condition is shown to have likely been racemose. The dichasial arrangement is associated with the branching potential of the flower rather than with the FSS, i.e. additional flowers emerge from the bracteole axils. Thus, the dichasium in *Eugenia* commonly referred to in taxonomic literature is better interpreted as an auxotelic inflorescence with dichasial arrangement and should be described accordingly.

## Conclusions

This study integrates inflorescence development and architecture within an evolutionary framework for Myrtaceae, providing new insights into inflorescence evolution in the hyper-diverse genus *Eugenia*. Previously overlooked features are shown to be relevant for systematics and taxonomy, supporting hypotheses of homology and highlighting that architectural flexibility is the rule rather than a fixed inflorescence model. Typological definitions should therefore incorporate developmental and evolutionary perspectives while remaining descriptive and practical. The use of terminology based on homologous inflorescence arrangements, complemented by detailed morphological descriptions, is recommended. Future studies on racemose branching patterns and on the mechanisms underlying architectural flexibility may clarify their role in species diversification and Neotropical evolution in *Eugenia*.

## Supplementary Material

plag037_Supplementary_Data

## Data Availability

Raw dataset and the R code used are available on Figshare at https://doi.org/10.6084/m9.figshare.33201315.
