# Supplementary material for: Inflorescence architecture and development in the megadiverse Eugenia (Myrteae, Myrtaceae): an evolutionary perspective
Source: AoB Plants. 2026 Aug 20;18(5):plag037. doi: 10.1093/aobpla/plag037 (PMC13551620; doi:10.1093/aobpla/plag037)

Table S1. Descriptions following previous studies of inflorescence arrangements in *Eugenia*.

Inflorescence arrangement	Description
Auxotelic shoot	An axillary branch may be described as auxotelic (Briggs and Johnson 1979) if the main axis is prolifically extended and maintains late vegetative growth potential in the apex from an active meristem (Prenner et al. 2009). Lateral flowers are subtended by foliose bracts providing a system equivalent to a flowering shoot system (Weberling 1988). Solitary flowers (sensu Landrum and Kawasaki 1997) in a broad sense including the subtended shoot can be also interpreted as auxotelic shoot with lateral flowers (see McVaugh 1956; Briggs and Johnson 1979). A related arrangement is the '*Stenocalyx* type' (McVaugh 1968; Barroso et al. 1991) in allusion to a genus currently treated within *Eugenia*.
Bracteate shoot	A flowering axis with scaly bracts subtending flowers and axes terminated by a flower or vegetative meristem (Landrum and Kawasaki 1997). The terms racemiform (e.g. Giaretta and Fraga 2014) or raceme (e.g. Mazine and Souza 2008, 2009) have been used as equivalents. The degree of morphological extremity of bracteose or foliose forms of the bracts (i.e. whether these structures are better referred to as bracts or leaves), determines the most appropriate terminology (McVaugh 1956).
Fascicle	A flowering axis with short internodes bearing pedicellate flowers subtended by scaly bracts. Fascicles have been related to bracteate shoots with reduced main axes (Landrum and Kawasaki 1997). Such arrangements are also termed fasciculiform (Sobral et al. 2012). When the main axis is even more reduced it is often called an umbel or said to be umbelliform (see McVaugh 1956, 1963) or umbel-like (Landrum 1991).
Glomerule	A flowering axis with short internodes bearing sessile flowers subtended by scaly bracts. Glomerules have been related to bracteate shoots where the main axes and pedicels are simultaneously reduced (Landrum and Kawasaki 1997). Flower with very short pedicels (subsessile) are also termed glomerule (e.g. Souza et al. 2016).
Dichasium	A solitary flower subtended by a pair of bracteoles from where a flower emerges from each axil and results in a three or more-flowered arrangement (McVaugh 1956, 1963); the middle flower is premature in development relative to the lateral ones. Successive repetition of the branching pattern to unspecified degrees, can result in compound-dichasia (see Souza & Morim 2008).
Thyrsoid	A main axis (first-order) that branches (second-order) prolifically in the lowermost portion, supporting compound-cymes (third-order or higher). Branching is gradually reduced to second-order towards the apex, giving a pyramidal appearance (see Endress 2010). Such an arrangement with racemose primary branching followed by a cymose secondary pattern is termed thyrsoid when indeterminate, i.e. terminated by a flower (Briggs & Johnson 1979).
Panicle	A main axis (first-order) that ends in a flower and subtends a series of lateral (second to further orders) axes also terminated by flowers (McVaugh 1956; Prusinkiewicz et al. 2007). This arrangement is unpredictable, and consequently typological definitions can be inapplicable, failing to incorporate all variation. The panicle can be compared to a thyrsoid with depauperate branching at the apex, averting an obvious cymose pattern. Some inflorescences cannot be assigned with confidence, either as derivations of racemose or cymose structures, due to the limitations of definition of these branching patterns (Endress 2010).

References

Barroso GM, Peixoto AL, Ichaso CLF, Costa CG, Guimarães EF, Lima HC. 1991. Myrtaceae. In: Sistemática de Angiospermas do Brasil. Viçosa: Universidade Federal de Viçosa, 377.

Briggs BG, Johnson LAS. 1979. Evolution in the Myrtaceae - Evidence from inflorescence structure. *Proceedings of The Linnean Society of New South Wales* **102**: 157–256.

Endress PK. 2010. Disentangling confusions in inflorescence morphology: Patterns and diversity of reproductive shoot ramification in angiosperms. *Journal of Systematics and Evolution* **48**: 225–239.

Giaretta A, Fraga CN. 2014. Two new *Eugenia* species (Myrtaceae) from the Brazilian Atlantic forest. *Phytotaxa* **163**: 113–120.

Landrum LR. 1991. A new genus of Myrtaceae (Myrtinae) from Mesoamerica. *Systematic Botany* **16**: 21–29.

Landrum LR, Kawasaki ML. 1997. The genera of Myrtaceae in Brazil: An illustrated synoptic treatment and identification keys. *Brittonia* **49**: 508–536.

Mazine FF, Souza VC. 2008. A new species of *Eugenia* (Myrtaceae) from north-eastern Brazil. *Botanical Journal of the Linnean Society* **158**: 775–777.

Mazine FF, Souza VC. 2009. New species of *Eugenia* sect. *Racemosae* (Myrtaceae) from Brazilian Amazon Rainforest. *Kew Bulletin* **64**: 147–153.

McVaugh R. 1956. Tropical American Myrtaceae: Notes on generic concepts and descriptions of previously unrecognized species. *Fieldiana: Botany* **29**: 145–228.

McVaugh R. 1963. Tropical American Myrtaceae II: Notes on generic concepts and descriptions of previously unrecognized species. *Fieldiana: Botany* **29**: 393–532.

McVaugh R. 1968. The Genera of American Myrtaceae - an Interim Report. *Taxon* **17**: 354–418.

Prenner G, Vergara-Silva F, Rudall PJ. 2009. The key role of morphology in modelling inflorescence architecture. *Trends in Plant Science* **14**: 302–309.

Prusinkiewicz P, Erasmus Y, Lane B, Harder LD, Coen E. 2007. Evolution and development of inflorescence architectures. *Science* **316**: 1452–1456.

Sobral M, Grippa CR, Souza MC, Aguiar OT, Bertoncello R, Guimarães TB. 2012. Fourteen new species and two taxonomic notes on Brazilian Myrtaceae. *Phytotaxa* **50**: 19–50.

Souza MC, Morim MP. 2008. Subtribos Eugeniinae O. Berg e Myrtinae O. Berg (Myrtaceae) na Restinga da Marambaia, RJ, Brasil. *Acta Botanica Brasilica* **22**: 652–683.

Souza MAD, Scudeller VV, Mendonça MS. 2016. Two new species of *Eugenia* (Myrtaceae) from Central Amazonia, Brazil. *Phytotaxa* **289**: 167–174.

Weberling F. 1988. The Architecture of Inflorescences in the Myrtales. *Annals of the Missouri Botanical Garden* **75**: 226–310.

Table S2. Herbarium samples and vouchers of mature material analysed.

Species	Collector	Number	Barcode	Collection
Eugenia neolatifolia Sobral & M.A.D.Souza	Forest Department	2412		K
Eugenia neolatifolia Sobral & M.A.D.Souza	N.Y. Sandwith	558		K
Eugenia neolatifolia Sobral & M.A.D.Souza	H.F. Talbot	s.n.		K
Eugenia neolatifolia Sobral & M.A.D.Souza	D.B. Fanshawe	22925		K
Eugenia neolatifolia Sobral & M.A.D.Souza	M. Hoff	6022		K
Eugenia neolatifolia Sobral & M.A.D.Souza	D. Toriola	1009		CAY
Eugenia acutata Miq.	F. Barros	2357		K
Eugenia acutata Miq.	J. Mattos	16387		K
Eugenia acutata Miq.	M. Verdi	6538		K
Eugenia acutata Miq.	A. Giaretta	1460		K
Eugenia acutata Miq.	P.L.R. Moraes	2494		RB
Eugenia belemitana McVaugh	U.N. Maciel	181		K
Eugenia belemitana McVaugh	A. Giaretta	1441		K
Eugenia belemitana McVaugh	T.N. Vasconcelos	s/n		K
Eugenia arvensis Vell.	S.A. Mori	12996		K
Eugenia arvensis Vell.	D.Sucre	7283		K
Eugenia arvensis Vell.	D. Sucre	5935		K
Eugenia arvensis Vell.	A.F. Vaz	952		RB
Eugenia arvensis Vell.	L.B. Pimentel		00837793	RB
Eugenia astringens Cambess.	F. Sellow	s/n	000276545	K
Eugenia astringens Cambess.	C. Farney	2342		K
Eugenia astringens Cambess.	C. Farney	4641		K
Eugenia astringens Cambess.	H.M. Dias	141		K
Eugenia astringens Cambess.	D. Sucre	2618		K
Eugenia aurata O.Berg	V.G. Staggemeier	500		K
Eugenia aurata O.Berg	V.G. Staggemeier	410		K
Eugenia aurata O.Berg	C.A.C. Ferreira	6376		K
Eugenia aurata O.Berg	F. Sellow	5637		K
Eugenia aurata O.Berg	J.A. Ratter	7982		K
Eugenia axillaris (Sw.) Willd.	E. Cabrera	8884		K
Eugenia axillaris (Sw.) Willd.	E. Matuda	16508		K
Eugenia axillaris (Sw.) Willd.	W.A. Schipp	825		K
Eugenia axillaris (Sw.) Willd.	P.C. Standley	56628		K
Eugenia axillaris (Sw.) Willd.	J.A. Ratter	6598		K
Eugenia azuruensis O.Berg	J.E.Q. Faria	2642		RB
Eugenia azuruensis O.Berg	J.E.Q. Faria	4186		RB
Eugenia azuruensis O.Berg	F.S. Araujo	1014		RB
Eugenia biflora (L.) DC.	M.A.D. Souza	76		K
Eugenia biflora (L.) DC.	C.N.Fraga	2918		K
Eugenia biflora (L.) DC.	D.J. Atkinson	53		K
Eugenia biflora (L.) DC.	G.S. Jenman	200		K
Eugenia biflora (L.) DC.	D.C. Daly	286		K
Eugenia bimarginata DC.	W.J. Burchell	5029		K

Eugenia bimarginata DC.	W.J. Burchell	4664		K
Eugenia bimarginata DC.	L. Kawasaki	1633		K
Eugenia bimarginata DC.	D.R. Hunt	5925		K
Eugenia bimarginata DC.	W. Ganev	1107		K
Eugenia brasiliensis Lam.			001018524	K
Eugenia brasiliensis Lam.			001018525	K
Eugenia brasiliensis Lam.	R.C. Forzza	4717		K
Eugenia brasiliensis Lam.	M. Peron	257		K
Eugenia brasiliensis Lam.	A.F. Vaz	698		K
Eugenia tenuipedunculata Kiaersk.	F. Barros	1759		K
Eugenia tenuipedunculata Kiaersk.	R. Reitz	9070		K
Eugenia tenuipedunculata Kiaersk.	J.M. Silva	1769		K
Eugenia tenuipedunculata Kiaersk.	F.F. Mazine	1010		K
Eugenia tenuipedunculata Kiaersk.	N.M. Ivanauskas	246		RB
Eugenia bullata Pancher ex Guillaumin	A. Guillaumin	7700		K
Eugenia bullata Pancher ex Guillaumin	H.S. Mackee	46038		P
Eugenia bullata Pancher ex Guillaumin	E. Vieillard	480	00543900	P
Eugenia bullata Pancher ex Guillaumin	J.P. Blanchon	196		P
Eugenia bullata Pancher ex Guillaumin	M. Balansa	87		P
Eugenia cerasiflora Miq.			001021737	K
Eugenia cerasiflora Miq.	G. Hatschbach	52165		K
Eugenia cerasiflora Miq.	R.M. Harley	22656		K
Eugenia cerasiflora Miq.	W. Ganev	2870		K
Eugenia cerasiflora Miq.	W. Ganev	3236		K
Eugenia coffeifolia DC.			000275728	K
Eugenia coffeifolia DC.	R.A.A. Oldeman	2562		K
Eugenia coffeifolia DC.	R.A.A. Oldeman	1671		K
Eugenia coffeifolia DC.			001018809	K
Eugenia coffeifolia DC.	L. Barrabe	236		K
Eugenia convexinervia D.Legrand	R. Reitz	4156		K
Eugenia convexinervia D.Legrand	R. Reitz	6323		K
Eugenia convexinervia D.Legrand	R. Reitz	6332		K
Eugenia convexinervia D.Legrand	F.F. Mazine	994		K
Eugenia convexinervia D.Legrand	J.M Silva	3890		K
Eugenia bunchosiifolia Nied.	A.P. Duarte	6055		RB
Eugenia bunchosiifolia Nied.	A. Giaretta	1021		RB
Eugenia bunchosiifolia Nied.	A. Giaretta	1312		RB
Eugenia bunchosiifolia Nied.	D. Folli	31		RB
Eugenia bunchosiifolia Nied.	J.G. Jardim	4332		RB
Eugenia dodonaeifolia Cambess.	Y. Mexia	4688		K
Eugenia dodonaeifolia Cambess.	E.B. Foresto	129		K
Eugenia dodonaeifolia Cambess.	S.V.A. Pessoa	807		K
Eugenia dodonaeifolia Cambess.	R. Marquete	4122		K
Eugenia dodonaeifolia Cambess.	F. Riedel	355		K
Eugenia dysenterica DC.	H.S. Irwin	5860		K
Eugenia dysenterica DC.	M.A. Silva	4455		K

Eugenia dysenterica DC.	J.A. Ratter	4410		K
Eugenia dysenterica DC.	E.P. Heringer	5289		K
Eugenia dysenterica DC.	A. Glaziou	21198		K
Eugenia dysenterica DC.	F.F. Mazine	466		K
Eugenia egensis DC.	C.R.D. Vieira	4895	00969783	RB
Eugenia egensis DC.	J.E.Q. Faria	4859		RB
Eugenia egensis DC.	L. Cayola	1413		RB
Eugenia egensis DC.	M. Lewis	40582		RB
Eugenia egensis DC.	M.G. Bovini	3074		RB
Eugenia excelsa O.Berg	B.A. Krukoff	1125		K
Eugenia excelsa O.Berg	R.S. Pinheiro	283		K
Eugenia excelsa O.Berg	A.F. Regnell	562		K
Eugenia excelsa O.Berg	D.S.D. Araujo	405		K
Eugenia excelsa O.Berg	J.G. Rando	152		RB
Eugenia excelsa O.Berg	E.J. Lucas	125		K
Eugenia expansa (O.Berg) Nied.	M.F. Santos	713		K
Eugenia expansa (O.Berg) Nied.	S.V.A. Pessoa	476		K
Eugenia expansa (O.Berg) Nied.	H.C. Lima	2265		K
Eugenia expansa (O.Berg) Nied.	G. Martinelli	10037		K
Eugenia expansa (O.Berg) Nied.	R. Guedes	2167		K
Eugenia flavescens DC.			000579518	K
Eugenia flavescens DC.	J.A. Ratter	4073		K
Eugenia flavescens DC.	G. Eiten	9109		K
Eugenia flavescens DC.	J.R. Grifford	G31	001021634	K
Eugenia flavescens DC.	N.T. Silva	1461		K
Eugenia florida DC.	K.M. Redden	4935		K
Eugenia florida DC.	R.T. Pennington	342		K
Eugenia florida DC.	R. Schnell	11754		K
Eugenia florida DC.	J.A. Ratter	1337		K
Eugenia florida DC.	G.C.G. Argent	6722		K
Eugenia florida DC.	E.P. Heringer	5463		K
Eugenia foetida Pers.	E. Cabrera	8768		K
Eugenia foetida Pers.	G.F. Gaumer	24123		K
Eugenia foetida Pers.	P.H. Gentle	1202		K
Eugenia foetida Pers.	W.A. Schipp	427		K
Eugenia foetida Pers.	F. Gaumer	s.n.	000276275	K
Eugenia goiapabana Sobral & Mazine	J.E.Q. Faria	2481		K
Eugenia goiapabana Sobral & Mazine	A.P. Fontana	5419		K
Eugenia goiapabana Sobral & Mazine	R.C. Forzza	4999		RB
Eugenia guanabarina (Mattos & D.Legrand) Giaretta & M.C.Souza	L.A. Mattos	614		K
Eugenia guanabarina (Mattos & D.Legrand) Giaretta & M.C.Souza	P. Duarte	6058		K
Eugenia guanabarina (Mattos & D.Legrand) Giaretta & M.C.Souza	K. Matsumoto	818		K
Eugenia guanabarina (Mattos & D.Legrand) Giaretta & M.C.Souza	L.A.M. Silva	2515		K

Eugenia guanabarina (Mattos & D.Legrand) Giaretta & M.C.Souza	A. Giaretta	1360	K
Eugenia handroi Mattos	G. Hatschbach	19555	RB
Eugenia handroi Mattos	P.C. Porto	743	RB
Eugenia handroi Mattos	J.E.Q. Faria	3100	RB
Eugenia handroi Mattos	N.M. Ivanauskas	6175	RB
Eugenia handroi Mattos	P.R.C. Farág	215	RB
Eugenia handroi Mattos	C. Farney	3924	RB
Eugenia involucrata DC.	L.O. Williams	7409	K
Eugenia involucrata DC.	E.M.N. Lughadha	222	K
Eugenia involucrata DC.	W.J. Burchell	5832	K
Eugenia involucrata DC.	C. Angeli	277	K
Eugenia involucrata DC.	M.F. Santos	732	K
Eugenia joseramosii M.A.D. Souza & Scudeller	J.E.L.S. Ribeiro	1767	K
Eugenia joseramosii M.A.D. Souza & Scudeller	M.A.D. Souza	155	K
Eugenia joseramosii M.A.D. Souza & Scudeller	M.A.D. Souza	248	K
Eugenia joseramosii M.A.D. Souza & Scudeller	A. Vicentini	1141	K
Eugenia joseramosii M.A.D. Souza & Scudeller	J.R. Nascimento	588	RB
Eugenia lagoensis Kiaersk.	C.N. Fraga	2436	K
Eugenia lagoensis Kiaersk.	D.C. Zappi	1843	RB
Eugenia lagoensis Kiaersk.	V.C. Souza	32896	RB
Eugenia lagoensis Kiaersk.	P.M. Andrade	644	RB
Eugenia melanogyna (D.Legrand) Sobral	F.F. Mazine	969	K
Eugenia melanogyna (D.Legrand) Sobral	J.M. Silva	3831	K
Eugenia melanogyna (D.Legrand) Sobral	G. hastschbach	44462	RB
Eugenia melanogyna (D.Legrand) Sobral	M.L. Brotto	1703	RB
Eugenia melanogyna (D.Legrand) Sobral	R. Reitz	4321	K
Eugenia modesta DC.	R.M. Harley	22975	K
Eugenia modesta DC.	M.L. Guedes	5658	K
Eugenia modesta DC.	M. Peron	594	K
Eugenia modesta DC.	T.N. Vasconcelos	476	K
Eugenia modesta DC.	F. Riedel	2589	K
Eugenia monticola (Sw.) DC.	R. Liesner	13243	MBM
Eugenia monticola (Sw.) DC.	C.L. Jones	129	MBM
Eugenia monticola (Sw.) DC.	R. Espinoza	824	K
Eugenia monticola (Sw.) DC.	Q. Jiménez	862	K
Eugenia monticola (Sw.) DC.	G. Rivera	1200	K
Eugenia monticola (Sw.) DC.	P. Sánchez	1299	K
Eugenia monticola (Sw.) DC.	W.C. Holmes	6630	MBM
Eugenia monticola (Sw.) DC.	C.L. Jones	129	MBM
Eugenia monticola (Sw.) DC.	R. Liesner	13243	MBM

Eugenia moschata (Aubl.) Nied. ex T.Durand & B.D.Jacks.	R. Spruce	1063		K
Eugenia moschata (Aubl.) Nied. ex T.Durand & B.D.Jacks.	R. Spruce	991		K
Eugenia moschata (Aubl.) Nied. ex T.Durand & B.D.Jacks.	C.A.C. Ferreira	7612		K
Eugenia moschata (Aubl.) Nied. ex T.Durand & B.D.Jacks.	R.A.A. Oldeman	3581		K
Eugenia moschata (Aubl.) Nied. ex T.Durand & B.D.Jacks.	A. Silva	227		K
Eugenia myrcianthes Nied.	F.C. Silva	s.n.	001178048	K
Eugenia myrcianthes Nied.	K. Fiebrig	34		K
Eugenia myrcianthes Nied.	B. Balansa	1322		K
Eugenia myrcianthes Nied.	J.R.I. Wood	26859		K
Eugenia myrcianthes Nied.	J.R.I. Wood	26896		K
Eugenia myrcianthes Nied.	H.A. Keller	5756		K
Eugenia subglomerata (Kuntze) Sobral	J.B.E. Pohl	1041		K
Eugenia subglomerata (Kuntze) Sobral	M. Glaziou	11999		K
Eugenia subglomerata (Kuntze) Sobral	A. Glaziou	7640		K
Eugenia subglomerata (Kuntze) Sobral	W.H. Stubbebine	13200		K
Eugenia subglomerata (Kuntze) Sobral	J.M.A. Braga	3089		K
Eugenia neograndifolia Mattos	G. Wachenheim	480		K
Eugenia neograndifolia Mattos	J.J. Granville	11207		K
Eugenia neograndifolia Mattos	J.J. Granville	4585		CAY
Eugenia neograndifolia Mattos	A. Giaretta	1616		K
Eugenia neograndifolia Mattos	T.R. van Andel	4676		K
Eugenia neograndifolia Mattos	B. Maguirre	23834		K
Eugenia neoverrucosa Sobral	E.J. Lucas	118		K
Eugenia neoverrucosa Sobral	F. Chagas	1411		K
Eugenia neoverrucosa Sobral	E. Lucas	132		K
Eugenia neoverrucosa Sobral	J.C. Lindeman	5837		K
Eugenia neoverrucosa Sobral	R. Reitz	3850		K
Eugenia nutans O.Berg	J.E.Q. Faria	2422		K
Eugenia nutans O.Berg	M.F. Santos	531		K
Eugenia nutans O.Berg	H.S. Irwin	19864		K
Eugenia nutans O.Berg	E. Lucas	260		K
Eugenia nutans O.Berg	J.E.Q. Faria	2464		K
Eugenia paludosa Pancher ex Brongn. & Gris	J.M. Veillon	8033		P
Eugenia paludosa Pancher ex Brongn. & Gris	H.S. Mackee	29306		P
Eugenia paludosa Pancher ex Brongn. & Gris	H.S. Mackee	29248		P
Eugenia paludosa Pancher ex Brongn. & Gris	H.S. Mackee	38796		P
Eugenia paludosa Pancher ex Brongn. & Gris	H.S. Mackee	5480		K
Eugenia patens Poir.	R.A.A. Oldeman	284		K

Eugenia patens Poir.	R.A.A. Oldeman	1888		K
Eugenia patens Poir.	R. Spruce	2261		K
Eugenia patens Poir.	W. Balée	2684		K
Eugenia patens Poir.	B.V. Rabelo	2403		K
Eugenia patens Poir.	I.L. Amaral	240		K
Eugenia pisiformis Cambess.	A. Glaziou	19364		K
Eugenia pisiformis Cambess.			001021558	K
Eugenia pisiformis Cambess.	S.J. Silva-Neto	457		K
Eugenia pisiformis Cambess.	A.C.S. Cavalcanti	265		k
Eugenia pisiformis Cambess.	R.O. Perdiz	911		RB
Eugenia pisiformis Cambess.	M.O. Bünger	562		RB
Eugenia pistaciifolia DC.	L.P. Queiroz	3839		K
Eugenia pistaciifolia DC.	L. Passos	5546		K
Eugenia pistaciifolia DC.	W. Ganev	1420		K
Eugenia pistaciifolia DC.	W. Ganev	1400		K
Eugenia pistaciifolia DC.	M.O.O. Pellegrini	359		RB
Eugenia pitanga (O.Berg) Nied.	F.F. Mazine	1020		K
Eugenia pitanga (O.Berg) Nied.	F.F. Mazine	1030		K
Eugenia pitanga (O.Berg) Nied.	C. Giraldi	s.n.	001018480	K
Eugenia pitanga (O.Berg) Nied.	T.M. Pedersen	6560		K
Eugenia pitanga (O.Berg) Nied.	E. Hassler	3218		K
Eugenia pluriflora DC.	W.J. Burchell	4930		K
Eugenia pluriflora DC.	J.C. Lindeman	1137		K
Eugenia pluriflora DC.	W.J. Burchell	4724		K
Eugenia pluriflora DC.	M. Glaziou	10806		K
Eugenia pluriflora DC.	L. Riedel	2186		K
Eugenia protenta DC.	R. Spruce	3816		K
Eugenia protenta DC.	M.A.D. Souza	97		K
Eugenia protenta DC.	M.A.D. Souza	99		K
Eugenia protenta DC.			000276652	K
Eugenia protenta DC.	A. Vicentini	1022		K
Eugenia punicifolia (Kunth) DC.	A.C. Smith	3352		K
Eugenia punicifolia (Kunth) DC.	B.A. Whitton	222		K
Eugenia punicifolia (Kunth) DC.	Gardner	4164		K
Eugenia punicifolia (Kunth) DC.	V. Staggemeier	302		K
Eugenia punicifolia (Kunth) DC.	F.F. Mazine	1065		K
Eugenia pyrifera Faria & Proença	J.E.Q. Faria	897		RB
Eugenia pyrifera Faria & Proença	J.E.Q. Faria	4122		RB
Eugenia pyrifera Faria & Proença	J.E.Q. Faria	2120		RB
Eugenia pyrifera Faria & Proença	J.E.Q. Faria	899		RB
Eugenia pyrifera Faria & Proença	J.E.Q. Faria	5178		RB
Eugenia pyriformis Cambess.	G. Eiten	6582		K
Eugenia pyriformis Cambess.	O. Handro	2093		K
Eugenia pyriformis Cambess.	G.F.J. Pabst	9185		K
Eugenia pyriformis Cambess.	G.Y. Saiki	s.n.	001018052	K
Eugenia pyriformis Cambess.	E. Hassler	5058		K

Eugenia pyriformis Cambess.	F.F. Mazine	1028		K
Eugenia reinwardtiana (Blume) DC.	Rahman	6759		K
Eugenia reinwardtiana (Blume) DC.	Backer	29415		K
Eugenia reinwardtiana (Blume) DC.	A. Mail	3910		K
Eugenia reinwardtiana (Blume) DC.	D.I. Nicholson	s.n.	001000274	K
Eugenia reinwardtiana (Blume) DC.	P. Orolfo	3793		K
Eugenia selloi B.D.Jacks.	A.Q. Lobão	358		K
Eugenia selloi B.D.Jacks.	D. Sucre	6054		K
Eugenia selloi B.D.Jacks.	M.O. Bünger	566		K
Eugenia selloi B.D.Jacks.	M.C. Souza	638		K
Eugenia selloi B.D.Jacks.	D. Sucre	9548		K
Eugenia sonderiana O.Berg	P.T. Sano	CFCR 14863	96432	SPF
Eugenia sonderiana O.Berg	M.A. Pena	802		SPF
Eugenia sonderiana O.Berg	F.F. Mazine	674		SPF
Eugenia sonderiana O.Berg	G. Hatschbach	41770		SPF
Eugenia sonderiana O.Berg	J.R. Stehmann	2316		SPF
Eugenia speciosa Cambess.	R.C. Forzza	4826		K
Eugenia speciosa Cambess.	P. Claussen	s.n.	001018490	K
Eugenia speciosa Cambess.			001018492	K
Eugenia speciosa Cambess.	E. Hassler	6259		K
Eugenia speciosa Cambess.	L.B. Smith	7256		K
Eugenia stipitata McVaugh	C.R. Sperling	6361		K
Eugenia stipitata McVaugh	B.A. Krukoff	6591		K
Eugenia stipitata McVaugh	G.T. Prance	5601		K
Eugenia stipitata McVaugh	C.N. Fraga	3188		RB
Eugenia stipitata McVaugh	V.G. Staggemeier	835		K
Eugenia subterminalis DC.	F.F. Mazine	974		K
Eugenia subterminalis DC.	A.A. Arantes	689		K
Eugenia subterminalis DC.	F.F. Mazine	1086		K
Eugenia subterminalis DC.	G. Hatschbach	61319		K
Eugenia subterminalis DC.	K. Fiebrig	100		K
Eugenia umbrosa O.Berg	M.F. Santos	792		K
Eugenia umbrosa O.Berg	M. Glaziou	8387		K
Eugenia umbrosa O.Berg	A. Glaziou	3008		K
Eugenia umbrosa O.Berg	A. Giaretta	1498		K
Eugenia umbrosa O.Berg	G. Martinelli	8488		K
Eugenia uniflora L.	F.C. Silva	1742		K
Eugenia uniflora L.	J.C. Lindeman	2076		K
Eugenia uniflora L.			001021251	K
Eugenia uniflora L.			001021242	K
Eugenia uniflora L.			001021240	K
Eugenia vattimoana Mattos	J. Miers	3802		K
Eugenia vattimoana Mattos	C. Angeli	191		K
Eugenia vattimoana Mattos	M.F. Gardner	5462		K
Eugenia vattimoana Mattos	R.N. Damasceno	1161		RB

Eugenia vattimoana Mattos	H.N. Braga	641		RB
Eugenia verticillata (Vell.) Angely	R. Reitz	6634		K
Eugenia verticillata (Vell.) Angely	G. hastschbach	14104		K
Eugenia verticillata (Vell.) Angely	J. Mattos	10825		K
Eugenia verticillata (Vell.) Angely	P. Claussen	107		K
Eugenia verticillata (Vell.) Angely	M. Glaziou	10790		K
Eugenia verticillata (Vell.) Angely	W.J. Burchell	3116		K
Eugenia wentii Amshoff	A. Prelle	351	001019880	K
Eugenia wentii Amshoff	B. Maguirre	24665		K
Eugenia wentii Amshoff	Boschwezen (BW)	6178	001019882	K
Eugenia wentii Amshoff	R. Evans	2712		K
Eugenia wentii Amshoff	M.F. Prévost	4705		K
Eugenia wentii Amshoff	S. Mori	22970		K
Eugenia wentii Amshoff	C.A.C. Ferreira	9286		K
Eugenia wentii Amshoff	L.O.A. Teixeira	604		K
Myrcianthes fragrans (Sw.) McVaugh	P.P. Moreno	22662		K
Myrcianthes fragrans (Sw.) McVaugh	G.B. Hinton	404		K
Myrcianthes fragrans (Sw.) McVaugh			000277416	K
Myrcianthes fragrans (Sw.) McVaugh			000261864	K
Myrcianthes fragrans (Sw.) McVaugh	G.P. DeWolf	160		
Myrcianthes pungens (O.Berg) D.Legrand	M. Lewis	40768		K
Myrcianthes pungens (O.Berg) D.Legrand	J.R.I. Wood	10521		K
Myrcianthes pungens (O.Berg) D.Legrand	J.C. Solomon	11080		K
Myrcianthes pungens (O.Berg) D.Legrand	J.M. Silva	6019		RB
Myrcianthes pungens (O.Berg) D.Legrand	V. Santos	77		RB
Myrtus communis L.	R.D. Kelly	29		K
Myrtus communis L.	G. Nicholson	s.n.		K
Myrtus communis L.	G. Nicholson	s.n.		K
Myrtus communis L.	N.D. Simpson	5332		K
Myrtus communis L.	J.J. Rein	54		K
Rhodamnia rubescens (Benth.) Miq.			00931	K
Rhodamnia rubescens (Benth.) Miq.	J. Garden	17246		K
Rhodamnia rubescens (Benth.) Miq.	A. Cunningham	s.n.		K
Rhodamnia rubescens (Benth.) Miq.	L.G. Adams	1632		K
Rhodamnia rubescens (Benth.) Miq.	A. Morrison	5599		K

Table S3. AIC values for character reconstruction models for combined five development patterns and panicle (ER = equal rates; ARD = all rates different; SYM = symmetrical; 'aic.w' is the computed AIC weights – see manual of phytools). Best AIC weights are indicated in bold.

		**ER**	**ARD**	**SYM**
**Branching patterns**	AIC	63,69426	68,27716	63,42705
	aic.w'	0,4456201	0,045061	**0,5093188**
**Inflorescence patterns**	AIC	196,9366	387,6172	228,0233
	aic.w'	**0,99999982**	0,00000000	0,00000018

Table S4. Transition rates between seven inflorescence patterns in Eugenia and panicle analysed independently. AIC values for character reconstruction models for eight development patterns analysed independently (ER = equal rates; ARD = all rates different; SYM = symmetrical).

10000 trees with a mapped discrete character with states:

a, f, g, l, p, r, t, u

a = auxotelic development patterns

f = fasciculiform development patterns

g - glomeruliform development patterns

l = auxotelic cataphylls development patterns

p = panicle development patterns

r = racemose development patterns

t - thyrsoid development patterns

u = umbelliform development patterns

trees have 31.8947 changes between states on average

changes are of the following types:

	a,f	a,g	a,l	a,p	a,r	a,t	a,u	f,a	f,g	f,l	f,p	f,r
x->y	5.6107	2.3789	3.5487	0.8468	1.3027	3.3243	1.46	1.0335	1.0141	0.2715	0.1429	0.1016

	f,t	f,u	g,a	g,f	g,l	g,p	g,r	g,t	g,u	l,a	l,f
x->y	0.2457	0.0746	0.3334	0.2163	0.3524	0.1115	0.0697	0.2088	0.0416	1.5664	0.2197

	l,g	l,p	l,r	l,t	l,u	p,a	p,f	p,g	p,l	p,r	p,t
x->y	0.8461	0.2737	0.1786	0.8491	0.1488	0.2952	0.1097	0.1276	0.2435	0.0695	0.2492

	p,u	r,a	r,f	r,g	r,l	r,p	r,t	r,u	t,a	t,f	t,g	t,l
x->y	0.0409	0.246	0.1103	0.1208	0.2248	0.106	0.2154	0.0397	0.8144	0.1383	0.1572	0.3672

	t,p	t,r	t,u	u,a	u,f	u,g	u,l	u,p	u,r	u,t
x->y	0.1714	0.108	0.0773	0.273	0.1076	0.1202	0.224	0.1061	0.0627	0.1966

mean total time spent in each state is:

	a	f	g	l	p	r
raw	431.2884758	101.2670739	23.19378430	140.165185	19.62455956	41.18503532
prop	0.5231363	0.1228331	0.02813317	0.170015	0.02380383	0.04995586

	t	u	total
raw	61.60397043	6.100405900	824.4285

prop 0.07472324 0.007399557 1.0000

	log(L)	d.f.	AIC	weight
ER.model_ptt	-101.31508	1	204.6302	9.999983e-01
ARD.model_ptt	-78.21334	56	268.4267	1.402033e-14
SYM.model_ptt	-87.60616	28	231.2123	1.689484e-06

Fig S1. Alternative reconstruction of ancestral states at nodes with according to the inflorescence development patterns interpreted independently with seven patterns (i.e. *auxotelic*, *auxotelic cataphylls*, *fasciculiform*, *glomeruliform*, *racemiform*, *thyrsoid*, *umbelliform*,) and panicle generated by aggregation of 10,000 stochastic character maps.

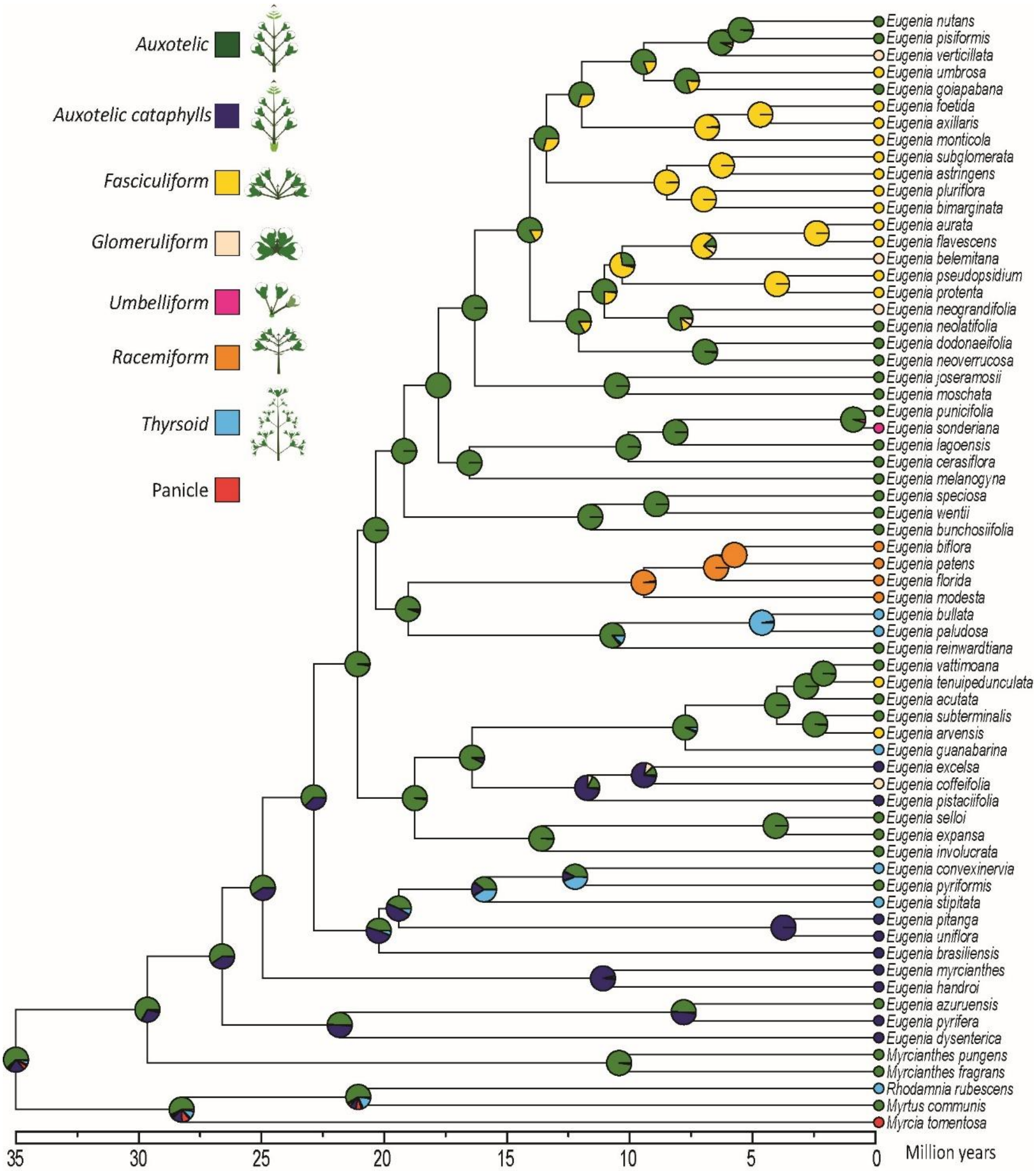

Supplement: Supplementary file 1 [file plag037_supplementary_data.pdf]
